# Tryptophan deficiency induced by indoleamine 2,3‐dioxygenase 1 results in glucose transporter 1‐dependent promotion of aerobic glycolysis in pancreatic cancer

**DOI:** 10.1002/mco2.555

**Published:** 2024-05-03

**Authors:** Heng Liang, Jiani Zhan, Yunqiu Chen, Zikang Xing, Zhen Ning Tony He, Yuying Liu, Xuewen Li, Yijia Chen, Zhiyao Li, Chunxiang Kuang, Dan Yang, Qing Yang

**Affiliations:** ^1^ State Key Laboratory of Genetic Engineering School of Life Sciences MOE Engineering Research Center of Gene Technology Shanghai Engineering Research Center of Industrial Microorganisms Fudan University Shanghai China; ^2^ Shanghai Key Lab of Chemical Assessment and Sustainability School of Chemical Science and Engineering Tongji University Shanghai China; ^3^ Department of Orthopedics Shanghai Children's Hospital School of Medicine Shanghai Jiao Tong University Shanghai China

**Keywords:** aerobic glycolysis, apoptosis, glucose transporter 1 (GLUT1), Indoleamine 2,3‐dioxygenase 1 (IDO1), pancreatic cancer

## Abstract

Indoleamine 2,3‐dioxygenase 1 (IDO1), the key enzyme in the catabolism of the essential amino acid tryptophan (Trp) through kynurenine pathway, induces immune tolerance and is considered as a critical immune checkpoint, but its impacts as a metabolism enzyme on glucose and lipid metabolism are overlooked. We aim to clarify the potential role of IDO1 in aerobic glycolysis in pancreatic cancer (PC). Analysis of database revealed the positive correlation in PC between the expressions of *IDO1* and genes encoding important glycolytic enzyme *hexokinase 2 (HK2)*, *pyruvate kinase (PK)*, *lactate dehydrogenase A (LDHA)* and *glucose transporter 1 (GLUT1)*. It was found that IDO1 could modulate glycolysis and glucose uptake in PC cells, Trp deficiency caused by IDO1 overexpression enhanced glucose uptake by stimulating GLUT1 translocation to the plasma membrane of PC cells. Besides, Trp deficiency caused by IDO1 overexpression suppressed the apoptosis of PC cells via promoting glycolysis, which reveals the presence of IDO1–glycolysis–apoptosis axis in PC. IDO1 inhibitors could inhibit glycolysis, promote apoptosis, and exhibit robust therapeutic efficacy when combined with GLUT1 inhibitor in PC mice. Our study reveals the function of IDO1 in the glucose metabolism of PC and provides new insights into the therapeutic strategy for PC.

## Graphical abstract

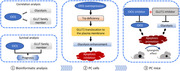

## 1 INTRODUCTION

Indoleamine 2,3‐dioxygenase 1 (IDO1) is the first and rate‐limiting enzyme catalyzing the breakdown of the essential amino acid tryptophan (Trp) through the kynurenine (Kyn) pathway to produce NAD^+^ and various bioactive intermediates, including Kyn. IDO1 can induce immune tolerance in cancer and is recognized as a critical immune checkpoint, alongside PD‐1 and CTLA‐4.^1^ Since IDO1's role in immune tolerance has been unveiled, piles of studies have focused on its role in cancer immunology, leading to underappreciation of the impacts of IDO1, the rate‐limiting enzyme involved in essential amino acid metabolism, on lipid and glucose metabolism.

A recent article demonstrates that in western diet‐induced metabolic syndrome, IDO1 inhibition positively regulated lipid metabolism, indicating the potential of IDO1 inhibitors in regulating metabolic disorders, such as obesity.^2^ The complicated role of IDO1 in atherosclerosis, a metabolic‐related disease marked by the excessive accumulation of lipids in the arteries,^3^ has also been revealed and discussed by our group and others.^4^, ^5^ Moreover, IDO1 enhances the fatty acid oxidation^6^, ^7^ and downregulates crucial enzymes engaged in T cells fatty acid synthesis.^8^ Nevertheless, only a limited body of studies concerns the effects of IDO1 on glucose metabolism, which are confined to glycolysis in T cells and embryonic stem cells.^7^, ^9^, ^10^, ^11^ The incidence of glycolysis in cancer cells is relatively high, but the effect of IDO1 on glycolysis in cancer cells is unclear.

Glycolysis is a universal pathway for the catabolism of glucose.^12^, ^13^ In most mammalian cells, glycolysis is inhibited by the presence of oxygen.^14^ One of the metabolic features of cancer cells is to dramatically increase their glycolytic flux even in the presence of oxygen—also known as the Warburg effect. The Warburg effect is a prominent metabolic process in cancer cells that affects cellular processes, fostering the uncontrolled growth and proliferation of cancer cells while inhibiting apoptosis.^15^ Existing evidence has indicated that the Warburg effect contributes greatly to tumor progression and could be a viable target for cancer therapy.^16^, ^17^ Glucose uptake is a rate‐limiting step in aerobic glycolysis in cancer cells, which is mediated by the glucose transporters (GLUT).^18^ There are 14 membrane‐bound proteins of the GLUT family, designated as GLUT1–GLUT14, of which GLUT1 is the most important and widely expressed family member.^19^ As it is frequently overexpressed in cancers and its involvement in the Warburg effect,^16^ GLUT1 is regarded as an antineoplastic target.^20^ Parsing the enzymatic function of IDO1 in cancer aerobic glycolysis is crucial and has the potential to deepen our understanding of IDO1's role in cancer.

Aerobic glycolysis is a hallmark of pancreatic cancer (PC), a malignant digestive tract cancer that is also considered a highly metabolic disease.^21^, ^22^, ^23^ PC is among the most aggressive and deadly forms of cancers, with a forecasted position as the second leading cause of cancer‐related fatalities in the United States in 2030.^24^ In PC cells, glucose uptake increases concurrently with the upregulation of GLUT1 expression and its translocation to the plasma membrane (PM).^16^, ^24^, ^25^, ^26^ Elevated expression of genes encoding rate‐limiting glycolytic enzymes, such as *hexokinase 2 (HK2)*, *phosphofructokinase 1*, and *lactate dehydrogenase A (LDHA)*, along with an increased glycolytic flux are frequently observed in PC.^24^ Aerobic glycolysis has been demonstrated to foster the growth and metastasis of PC, indicating its potential as a therapeutic target. Therapies for PC targeting altered glycolysis pathways are gaining momentum.^24^

Herein, we would like to clarify the potential role of IDO1 in aerobic glycolysis in PC. In this study, the correlations between the expressions of *IDO1* and genes encoding rate‐limiting glycolytic enzymes such as *HK2*, *PK*, *LDHA*, and *GLUT* family members in PC patients were analyzed with bioinformatic tools, and the regulatory effects of IDO1 on glycolysis as well as on glucose uptake were explored in mouse and human PC cells. The effects of IDO1 on GLUT1 expression and translocation to the PM of PC cells were investigated. Besides, the effect of IDO1‐regulated glycolysis on the proliferation and apoptosis of PC cells was investigated. The glycolysis inhibitory effect and apoptosis promoting effect of IDO1 inhibitors (RY103, INCB024360, and 1‐methyl‐l‐tryptophan [1‐MT]) were elucidated in *vivo* by using orthotopic and tumor‐bearing PC mice, and the therapeutic efficacy of IDO1 and GLUT1 inhibitors combination against PC was evaluated. This study may reveal new insight into the pathogenesis and therapeutic strategy for PC.

## 2 RESULTS

### 2.1 Overexpression of Trp‐catabolizing enzyme IDO1 in PC cells promotes glycolysis

**FIGURE 1:**
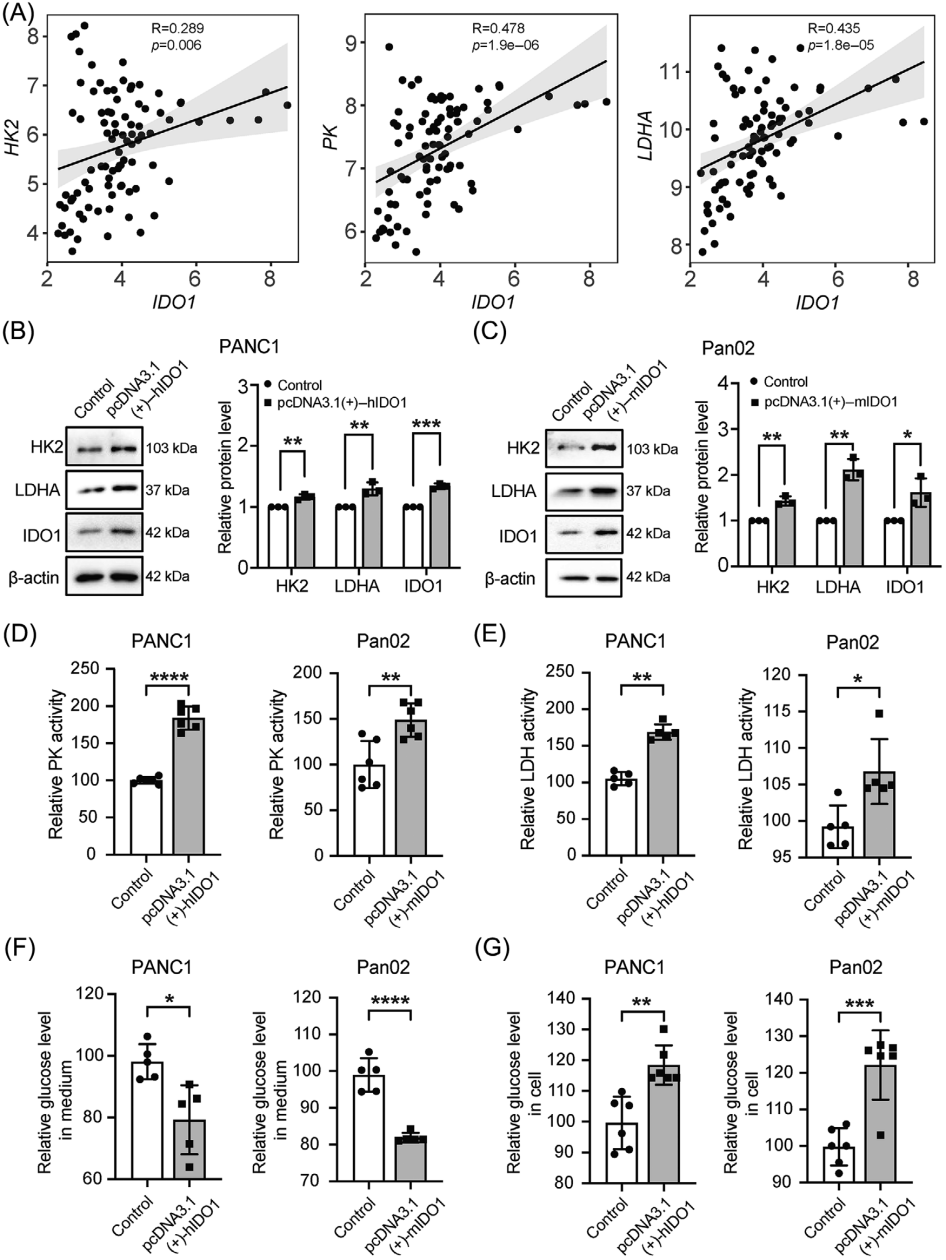
Overexpression of IDO1 in PC cells promotes glycolysis. (A) Scatter plot depicting the correlation of *IDO1* expression with expression of genes encoding the rate‐limiting glycolytic enzyme *HK2*, *PK*, and *LDHA* in human PC per GSE28735 dataset microarray gene expression profile data, *n* = 90. (B–G) PANC1 and Pan02 cells were transfected with IDO1 expressing plasmids and then cultured for 72 and 48 h, respectively. (B and C) Protein expression levels of HK2, LDHA, and IDO1 in PC cells detected by Western blot, *n* = 3. (D) PK activity in PC cells analyzed by colorimetric assay, *n* = 6. (E) LDH activity in PC cells analyzed by colorimetric assay, *n* = 5. (F) Glucose level in PC cell culture medium analyzed by colorimetric assay, *n* = 5. (G) Glucose level in PC cells analyzed by colorimetric assay, *n* = 6. Data were analyzed by the Student's *t*‐test and expressed as mean ± SD, *p < 0.05, **p < 0.01, ***p < 0.001, ****p < 0.0001.

The effect of Trp‐catabolizing enzyme IDO1 on glycolysis in cancers has not been explored and warrants clarification. Analysis of the GSE28735 dataset from the Gene Expression Omnibus (GEO) database revealed the positive correlation in PC between the expression of *IDO1* and the genes encoding the rate‐limiting glycolytic enzyme *HK2*, *pyruvate kinase (PK)*, and *LDHA* (Figure 1A). To explore the potential regulatory effect of IDO1 on glycolysis in PC, IDO1 was overexpressed either stably (Figure S1A) or transiently (Figures 1B, C, and S1B), in human PANC1 cells and mouse Pan02 cells. Results showed that transient overexpression of IDO1 yielded by transfecting IDO1 expressing plasmid for 48 h upregulated the expression of HK2 and LDHA in Pan02 cells (Figure 1C), but not significantly in PANC1 cells (Figure S1B). Interestingly, while we prolonged the culture time of PANC1 cells after IDO1 expressing plasmid transfection to 72 h, the expressions of HK2 and LDHA were significantly upregulated (Figure 1B). It seemed that transfecting IDO1 expressing plasmid for 72 h in PANC1 cells produced a more remarkable effect on glycolysis. Therefore, our following experiments were conducted under this condition: the culture time of Pan02 and PANC1 cells after IDO1 expressing plasmid transfection were 48 and 72 h, respectively. Further, we found that transient overexpression of IDO1 enhanced the activity of PK and lactate dehydrogenase (LDH) in PC cells (Figures 1D and E). Besides, we investigated the effect of IDO1 on glucose uptake, a rate‐limiting step of glycolysis, in PC cells. It was found that overexpression of IDO1 increased the glucose levels in PC cells and decreased the glucose levels in the cell culture medium of PC cells (Figures 1F and G), which suggested that overexpression of IDO1 promoted glucose uptake in PC cells. With IDO1 stable overexpressing PANC1 and Pan02 cells, we also found that overexpression of IDO1 enhanced the activity of LDH, increased the glucose levels in the cells, and decreased the glucose levels in the cell culture medium (Figures S1C–E). These results indicated that overexpression of IDO1 in PC cells promoted glycolysis.

### 2.2 Trp deficiency caused by IDO1 overexpression enhances glucose uptake in PC cells

**FIGURE 2:**
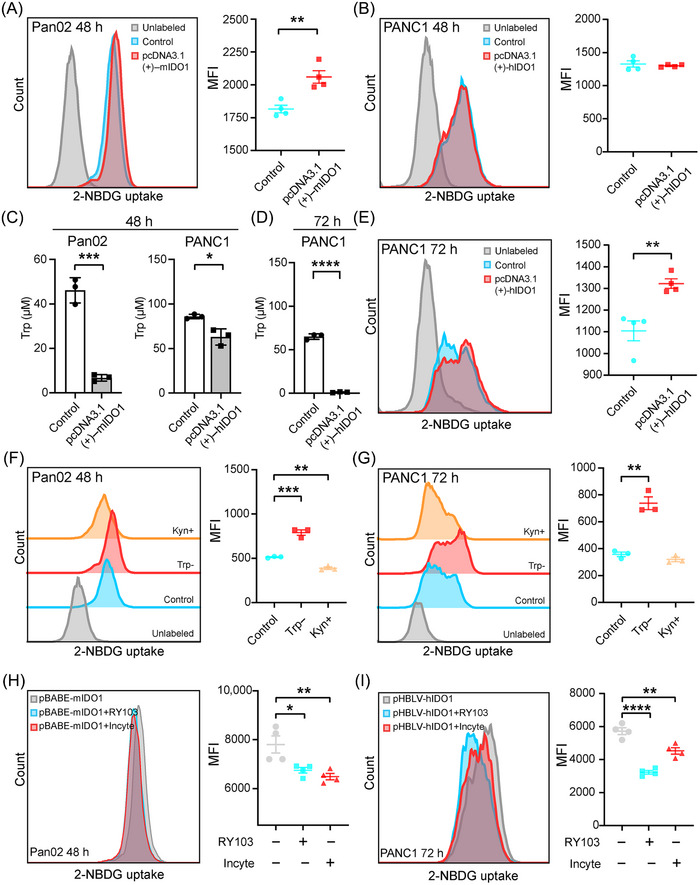
Trp deficiency caused by IDO1 overexpression enhances glucose uptake in PC cells. (A–E) Effect of IDO1 overexpression on glucose uptake and Trp level in PC cells. PC cells were transfected with IDO1 expressing plasmids and then cultured for 48 h (A–C) or 72 h (D and E). (A, B, and E) Glucose uptake determined using 2‐NBDG by flow cytometry. *n* = 4. (C and D) Trp levels in the supernatant of PC cells analyzed by HPLC, *n* = 3. (F and G) Effects of Trp and Kyn on glucose uptake in PC cells. Trp‐: Pan02 and PANC1 cells were cultured with Trp‐deficient medium for 48 and 72 h, respectively. Kyn+: Pan02 and PANC1 cells were cultured with DMEM medium supplemented with 100 µM Kyn for 48 h and 72 h, respectively. Glucose uptake determined using 2‐NBDG by flow cytometry, *n* = 3. (H and I) Effects of IDO1 inhibitors on glucose uptake in PC cells. IDO1 stable overexpressing Pan02 cells (pBABE–mIDO1) and PANC1 cells (pHBLV–hIDO1) were supplemented with RY103 (500 nM) or INCB024360 (Incyte, 500 nM) and cultured with DMEM medium for 48 and 72 h, respectively. Glucose uptake in PC cells determined using 2‐NBDG by flow cytometry. *n* = 4. Glucose uptake assays were conducted three times independently, and one representative data were shown. Data in panels (A)–(E) were analyzed by the Student's *t*‐test, and the other data were analyzed by one‐way ANOVA followed by Dunnett's post hoc test and expressed as mean ± SD, *p < 0.05, **p < 0.01, ***p < 0.001, ****p < 0.0001.

To validate the impact of IDO1 overexpression on glucose uptake, we conducted the glucose uptake assay by exposing PC cells to the fluorescent glucose analog 2‐(N‐(7‐nitrobenz‐2‐oxa‐1,3‐diazol‐4‐yl) amino)‐2‐deoxy‐d‐glucose (2‐NBDG), and subsequently detecting the fluorescence produced by the cells using flow cytometry.^27^ It was found that transient overexpression of IDO1 significantly enhanced glucose uptake in Pan02 cells, but not in PANC1 cells (Figures 2A and B). To find out the reason behind this, we analyzed the IDO1 activity in the cell culture medium of Pan02 and PANC1 cells by detecting the concentrations of Trp and Kyn. It was found that transient overexpression of IDO1 yielded by transfecting IDO1 expressing plasmid for 48 h increased IDO1 activity (decreasing Trp level and increasing Kyn level) more significantly in Pan02 than in PANC1 cells, and the Trp deficiency in Pan02 cells was remarkable than in PANC1 cells (Figures 2C and S2A, B). Specifically, upon transfection of IDO1 expressing plasmid for 48 h, Trp level in Pan02 cells was less than 10 µM, but was around 60 µM in PANC1 cells (Figure 2C). Then, we tried to induce enough Trp deficiency in PANC1 cells by prolonging the cell culture time post IDO1 expressing plasmid transfection to 72 h (Figures 2D and S2C), and as expected found that Trp concentration was decreased to below 10 µM (Figure 2D) and the glucose uptake was enhanced in PANC1 cells (Figure 2E). To confirm the function of Trp deficiency on glucose uptake, we used the Trp‐deficient cell culture medium (see *Materials and Methods*) to investigate the impact of Trp deficiency on glucose uptake. Results showed that Trp deficiency enhanced glucose uptake in Pan02 and PANC1 cells (Figures 2F and G), but the supplementation of Kyn, the Trp metabolite, did not enhance the glucose uptake in PC cells (Figures 2F and G). In addition, we found that short‐term (6 h) Trp deficiency enhanced glucose uptake and glucose levels in PC cells (Figures S2D, E and H). Further, in IDO1 stable overexpressing PC cells, glucose uptake was also enhanced (Figures S2F and G), and our self‐developed IDO1 inhibitor RY103^28^ and IDO1 inhibitor INCB024360 (Incyte)^29^ reversed the enhanced glucose uptake (Figures 2H and I). These results revealed that Trp deficiency caused by IDO1 overexpression enhanced glucose uptake in PC cells.

### 2.3 Trp deficiency caused by IDO1 overexpression stimulates GLUT1 translocation to the PM of PC cells

**FIGURE 3:**
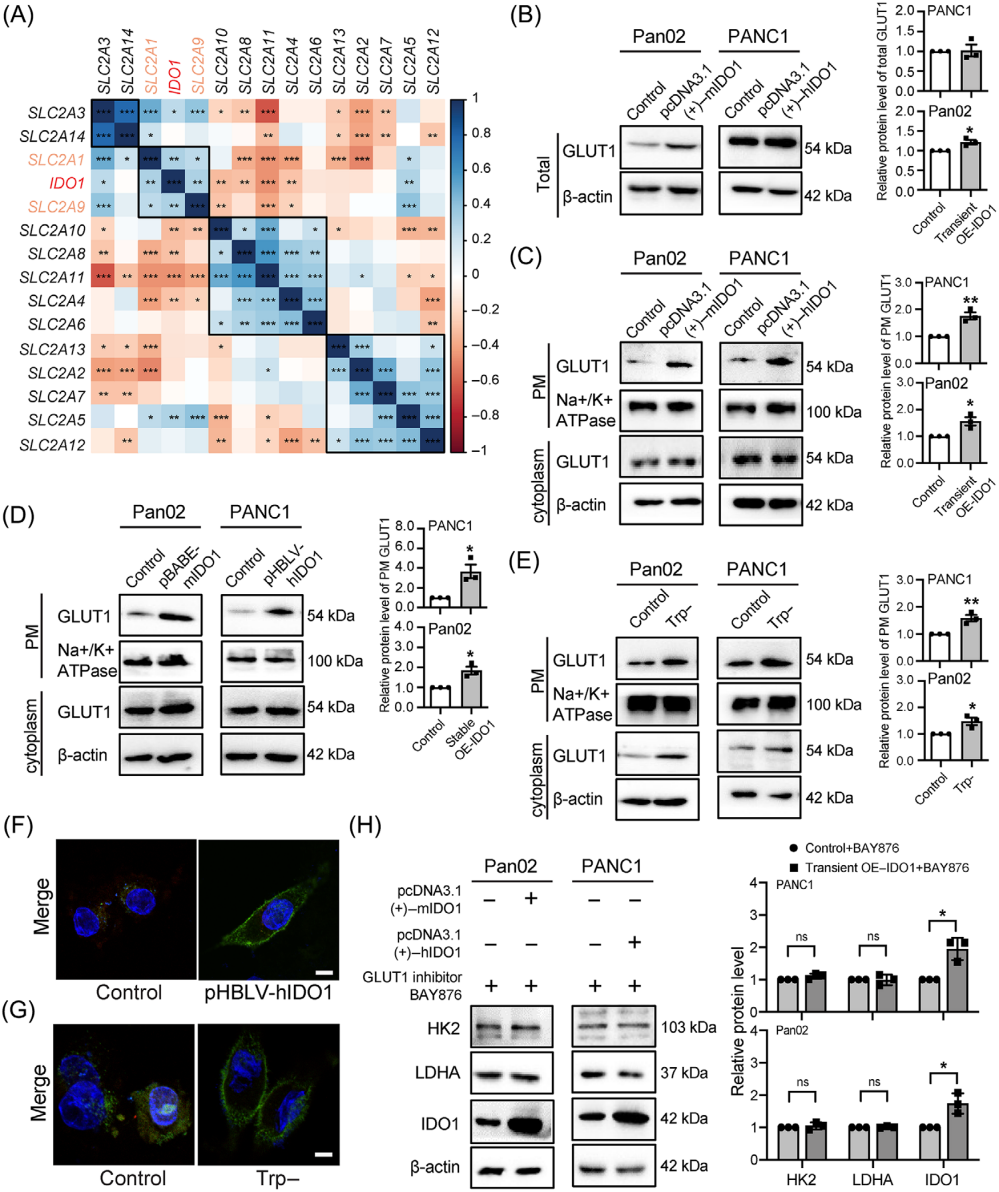
Trp deficiency caused by IDO1 overexpression stimulates GLUT1 translocation to the PM of PC cells. (A) Matrix graph of Pearson correlations of *IDO1* and SLC2A family gene mRNA expression levels in GSE28735, *n* = 90. (B–E) Effect of IDO1 overexpression and Trp deficiency on the protein expression of total, PM and cytoplasm GLUT1 in PC cells detected by Western blot accompanying quantification, *n* = 3. β‐Actin was used as a loading control for total and cytoplasm protein, and Na+/K+ ATPase was used as a loading control for PM protein. (B and C) PC cells were transfected with IDO1 expressing plasmids then cultured for 48 h (Pan02) or 72 h (PANC1). (D) IDO1 stable overexpressing PC cells were cultured for 48 h (Pan02) or 72 h (PANC1). (E) Trp‐: PC cells were cultured with Trp‐deficient medium for 6 h. (F and G) Effect of IDO1 overexpression and Trp deficiency on GLUT1 translocation to the PM analyzed by GLUT1 tandem fluorescence tracing system. The nucleus was stained with DAPI (Blue) before observation. Scale bar: 10 µm. (F) IDO1 stable overexpressing PANC1 cells were transfected with the mCherry–EGFP–GLUT1 then cultured for 72 h. (G) Trp‐: PANC1 cells were transfected with the mCherry–EGFP–GLUT1, cultured for 48 h, then incubated in Trp‐deficient medium for 6 h. (H) PC cells were transfected with IDO1 expressing plasmids and then cultured for 48 h (Pan02) or 72 h (PANC1), both receiving 1 µM BAY876 treatment 24 h before the experiments ended. Protein expression levels of HK2, LDHA, and IDO1 in PC cells detected by Western blot, *n* = 3. The data in panels (B)–(E), and (H) were analyzed by the Student's *t*‐test and expressed as mean ± SD, ns: not significant, *p < 0.05, **p < 0.01, ***p < 0.001.

The transport of glucose across the PM of cells is mediated by GLUT.^16^ To clarify the mechanism behind the IDO1‐overexpression‐enhanced glucose uptake in PC cells, we calculated the correlation between the gene expression of *IDO1* and the 14 members of the *solute carrier 2A (SLC2A)* family (encoding GLUT proteins)^30^ in PC patients using the GEO database. Results showed that the gene expression of *IDO1* is positively correlated with the gene expression of *SLC2A1* (encoding GLUT1 protein) and *SLC2A9* (encoding GLUT9 protein) in PC patients (Figure 3A). It is established that proliferating cells primarily rely on GLUT1 for glucose uptake.^31^ Notably, GLUT1 is upregulated in PC, and its high expression positively correlates with adverse clinical outcomes in PC patients. On the other hand, the function of GLUT9 has received little attention, and no report on the correlation between GLUT9 and PC pathogenesis has been found. So, we mainly focused on the contribution of GLUT1 in IDO1‐overexpression‐enhanced glucose uptake in PC cells in this study. We first found that transient overexpression of IDO1 upregulated GLUT1 expression in Pan02 cells but not significantly in PANC1 cells (Figure 3B). It is firmly established that enhanced translocation of GLUT1 to the PM actively facilitates glucose uptake.^16^ Intriguingly, we next found that transient or stable overexpression of IDO1 in PC cells promoted GLUT1 translocation to the PM by significantly increasing the GLUT1 levels on the PM but not changing the GLUT1 levels in the cytoplasm (Figures 3C, D, and S3A, B). Besides, GLUT1 translocation to the PM in PC cells cultured with Trp‐deficient medium was remarkably promoted by significantly increasing the GLUT1 levels both on PM and cytoplasm (Figures 3E and S3C). Using a tandem fluorescence tracing system of GLUT1,^31^ we also found that overexpression of IDO1 or Trp deficiency promoted GLUT1 translocation to the PM in PANC1 cells (Figures 3F and G). Similar results of IDO1 overexpression promoted GLUT1 translocation to the PM in Pan02 cells were also observed by immunofluorescence staining (Figure S3D). These results revealed that Trp deficiency caused by IDO1 overexpression promoted GLUT1 translocation to the PM in PC cells. Moreover, we found that the upregulation of HK2 and LDHA expression induced by IDO1 overexpression was significantly mitigated with the addition of the GLUT1 inhibitor BAY876 (Figures 1B, C and 3H), which implies that increased glycolysis induced by IDO1 overexpression is GLUT1‐dependent.

### 2.4 Trp deficiency caused by IDO1 overexpression inhibits apoptosis of PC cells via promoting glycolysis

**FIGURE 4:**
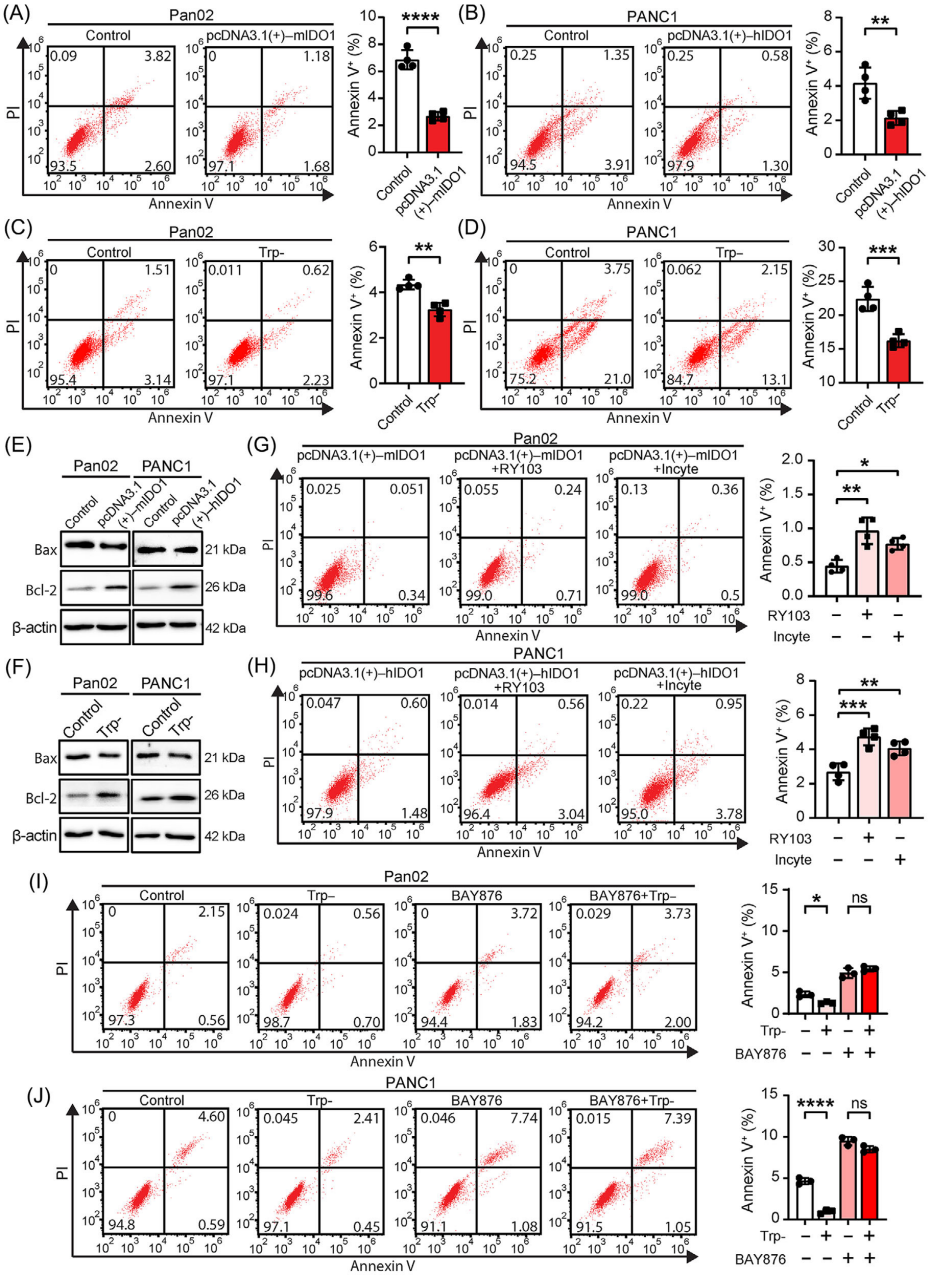
Trp deficiency caused by IDO1 overexpression inhibits apoptosis. (A–D) Effect of IDO1 overexpression and Trp deficiency on PC cell apoptosis. Apoptotic cells were determined by flow cytometry with staining of AnnexinV/PI, *n* = 4. (A and B) PC cells were transfected with IDO1 expressing plasmids then cultured for 48 h (Pan02) or 72 h (PANC1). (C and D) Trp‐: PC cells were cultured with Trp‐deficient medium for 6 h. (E and F) Protein expressions of Bax and Bcl‐2 in PC cells were detected by Western blot. (G and H) Effects of IDO1 inhibitors on IDO1 overexpression decreased cell apoptosis. PC cells were transfected with IDO1 expressing plasmids then cultured with DMEM medium supplemented with RY103 (500 nM) or Incyte (500 nM) for 48 h (Pan02) and 72 h (PANC1), respectively. Apoptotic cells were determined by flow cytometry with staining of AnnexinV/PI, *n* = 4. (I and J) Effects of glycolysis inhibitor BAY876 on Trp deficiency decreased cell apoptosis. PC cells were pretreated with 1 µM BAY876 for 12 h, then incubated in Trp‐deficient DMEM medium supplemented with 1 µM BAY876 for 6 h. Apoptotic cells determined by flow cytometry with staining of AnnexinV/PI, *n* = 3. Data in panels (A)–(D) were analyzed by the Student's *t*‐test, and data in panels (G)–(J) were analyzed by one‐way ANOVA followed by Dunnett's post hoc test and expressed as mean ± SD, ns: not significant, *p < 0.05, **p < 0.01, ***p < 0.001, ****p < 0.0001.

The data from Figures 1, 2, 3 demonstrated that IDO1 overexpression promoted the glycolysis of PC cells via inducing Trp deficiency and then promoting the GLUT1 translocation to the PM. Aerobic glycolysis is a major metabolic process in cancer cells that influences cellular functions, creating conditions favorable for uncontrolled proliferation and the inhibition of apoptosis in cancer cells.^15^, ^32^, ^33^ It is worth exploring whether IDO1‐regulated glycolysis affects the proliferation and apoptosis of PC cells. First, we found that transient overexpression of IDO1 did not change the proliferation (Figure S4A) but inhibited the apoptosis of PC cells (Figures 4A and B). Apoptosis was also inhibited in IDO1 stable overexpressing Pan02 and PANC1 cells (Figures S4B and C). Next, we found that Trp deficiency suppressed the apoptosis of PC cells (Figures 4C and D). Also, the results of Western blot showed that transient and stable overexpression of IDO1 and Trp deficiency in PC cells down regulated the expression of Bax (proapoptosis protein^34^) and upregulated the expression of Bcl‐2 (antiapoptosis protein^34^) (Figures 4E, F and S4D, E). We then examined whether the inhibitory effect of IDO1 overexpression on apoptosis of PC cells could be interfered by IDO1 inhibitors. As shown in Figures 4G and H, IDO1 inhibitor RY103 or Incyte significantly restored the decreased apoptosis of PC cells caused by IDO1 overexpression. Similar to our result, it has been reported that IDO1 overexpression was linked to the reduction of apoptosis of endometrial stromal or breast cancer cells^35^, ^36^ and IDO1 inhibitor Incyte markedly promoted apoptosis of oral squamous cell carcinoma or colon cancer cells both in vitro and in vivo.^37^, ^38^ Further, we used the selective GLUT1 inhibitor BAY876, which is also a potent blocker of glycolysis,^39^ to confirm the role of glycolysis in the decreased apoptosis of PC cells caused by Trp deficiency. The result showed that BAY876 abolished the suppression effect of Trp deficiency on apoptosis in PC cells (Figures 4I and J). Together with the above‐mentioned data, these results demonstrated that IDO1‐overexpression‐induced Trp deficiency promoted glycolysis and decreased the apoptosis of PC cells, and the suppression of Trp deficiency on apoptosis was abolished when glycolysis was inhibited. The result reveals the presence of the IDO1–glycolysis–apoptosis axis in PC for the first time.

### 2.5 IDO1 inhibitors downregulate glycolytic enzyme expression in tumors and reduce LDH levels in the serum of orthotopic PC mice

**FIGURE 5:**
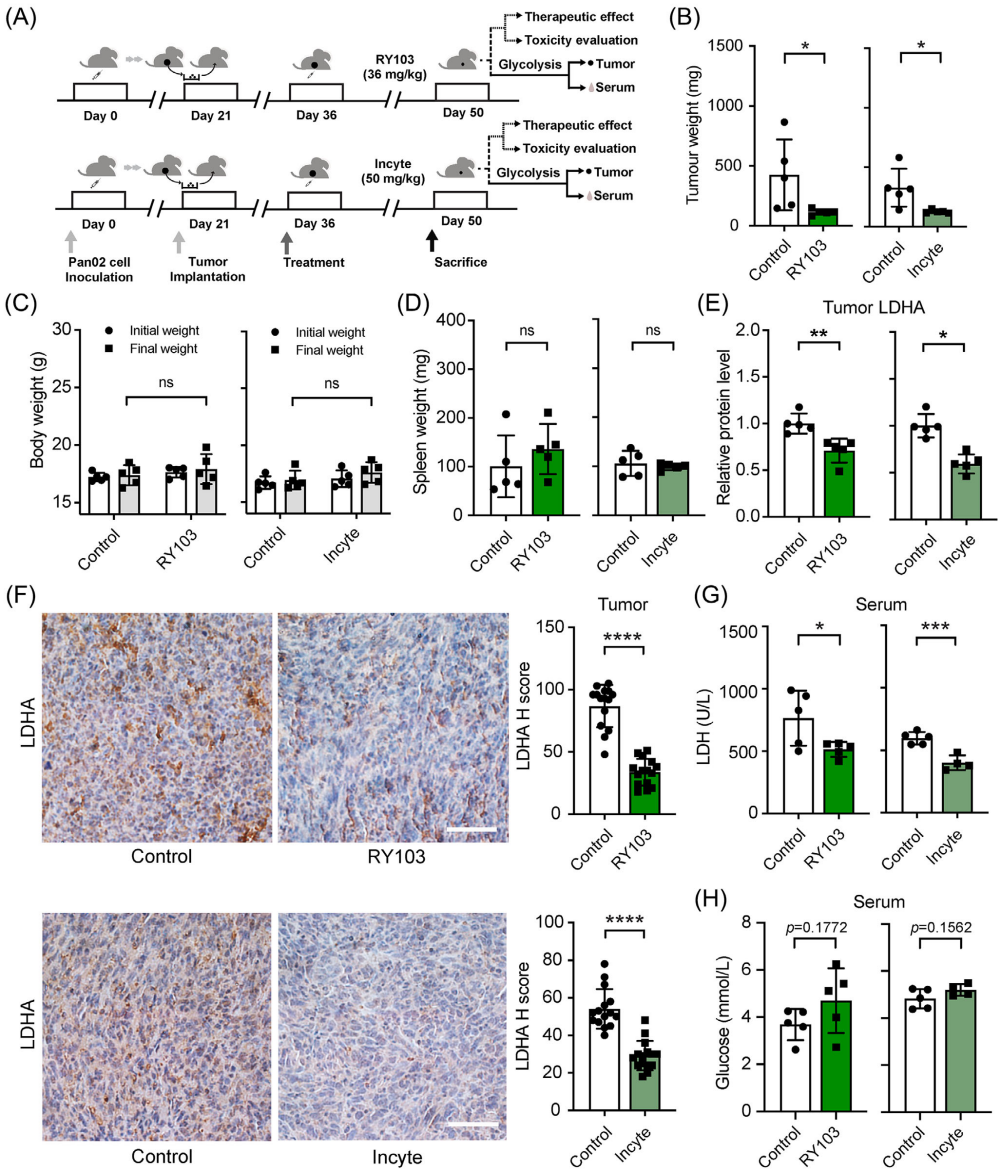
IDO1 inhibitors downregulate glycolytic enzyme expression in tumors and reduce LDH levels in the serum of orthotopic PC mice. Pan02 orthotopic PC mice were administered with RY103 (36 mg/kg, i.p., every 36 h) and Incyte (50 mg/kg, i.p., every 24 h) for 2 weeks and sacrificed 1 day post the last administration. (A) Graphical representation outlining the treatment schedule. (B–D) Tumors and spleens isolated and weighed. Body weight measured at the initiation and completion of treatments, *n* = 5/group. (E) Protein expression levels of LDHA in tumor tissues of Pan02 orthotopic PC mice detected by Western blot, *n* = 5/group. (F) Immunohistochemistry analysis of the expression of LDHA in tumor tissues from 15 sections from 5 mice/group and the scale bar is 50 µm. (G and H) LDH activities and glucose levels in serum of Pan02 orthotopic PC mice analyzed by ADVIA XPT, Control: *n* = 5/group, RY103: *n* = 5/group, Incyte: *n* = 4/group. Data were analyzed by the Student's *t*‐test and expressed as mean ± SD, ns: not significant, *p < 0.05, **p < 0.01, ***p < 0.001, ****p < 0.0001.

To examine whether IDO1 inhibitors could inhibit glycolysis in *vivo*, we constructed the Pan02 orthotopic PC mice (Figure 5A). It was found that IDO1 inhibitor RY103 or Incyte inhibited tumor growth (Figures 5B, S5A, and S6A). Both of the inhibitors did not impact the body weight and spleen weight of the mice, which indicated that the IDO1 inhibitors had no apparent toxicity (Figures 5C and D). Besides, we also found that long‐term administration of RY103 significantly suppressed tumor growth and had no apparent toxicity (Figures S7A–D). IDO1 inhibitor RY103 or Incyte significantly suppressed the expression of LDHA in tumors of the PC mice and the serum LDH levels (Figures 5E–G, S5B, and S6B), and slightly increased the glucose levels in the serum (Figure 5H), but did not affect the cholesterol, high‐density lipoprotein (HDL) cholesterol, low‐density lipoprotein (LDL) cholesterol, and triglycerides levels (Figures S5C–F and S6C–F), which indicated that IDO1 inhibitors inhibited glycolysis in *vivo* but did not affect the lipid metabolism. Low LDH levels in serum significantly correlated with longer overall survival (OS) of PC patients,^40^, ^41^, ^42^, ^43^ which suggests RY103‐ or Incyte‐treated mice with low serum LDH levels may have a longer survival time than the control mice. Indeed, our previous study had shown that IDO1 inhibitor RY103 prolonged the survival time of the orthotopic PC mice.^28^

### 2.6 Combination of IDO1 inhibitor with GLUT1 inhibitor exhibits robust therapeutic efficacy in PC mice

**FIGURE 6:**
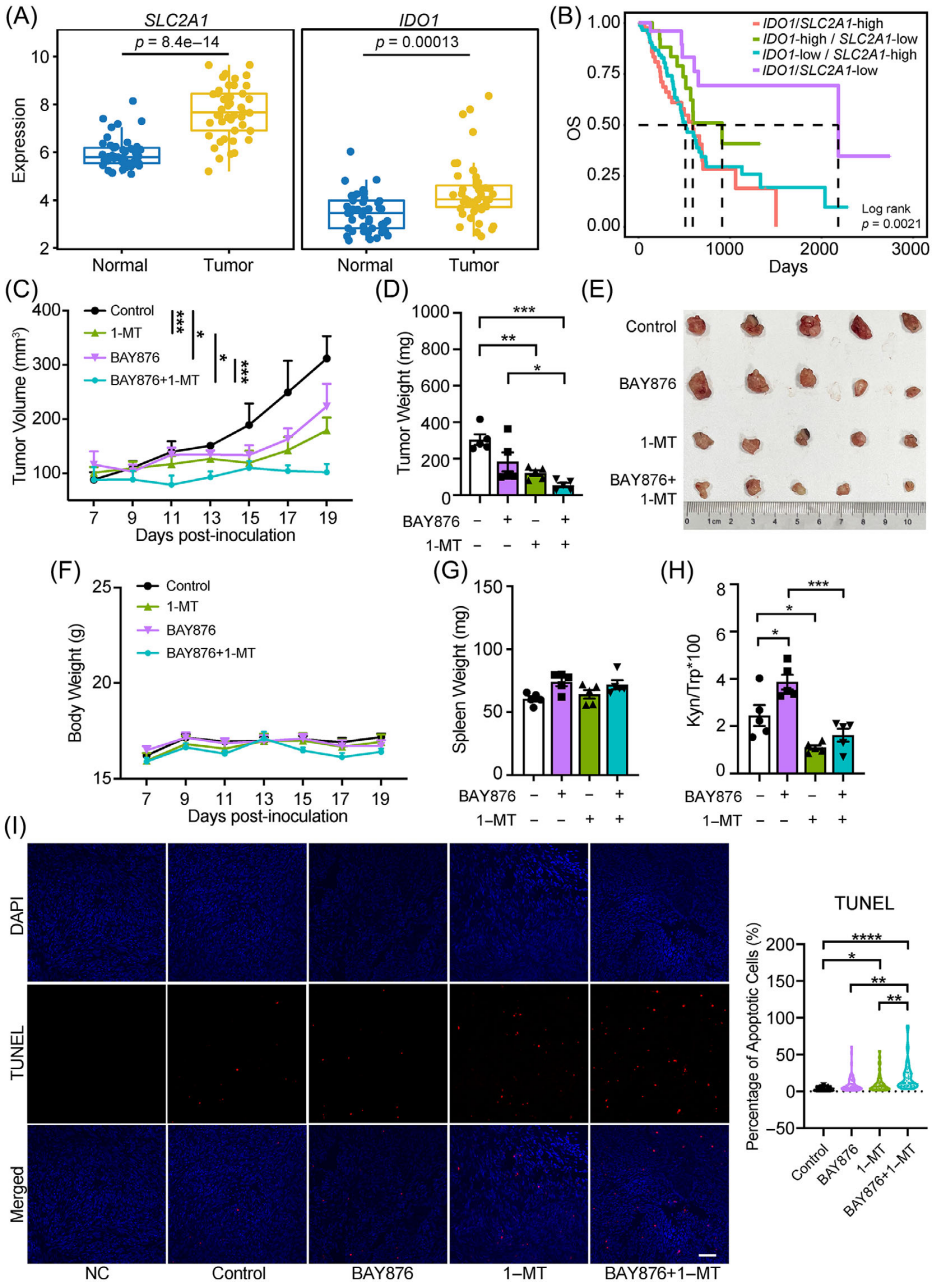
IDO1 inhibitor exhibit robust therapeutic efficacy combined with GLUT1 inhibitor in PC mice. Pan02 tumor‐bearing mice were administered with 1‐MT (100 mg/kg, i.p., every 36 h) and/or BAY876 (2.5 mg/kg, i.g., every 24 h) for 2 weeks and sacrificed 1 day post the last administration. (A) Gene expression levels of *SLC2A1* and *IDO1* in tumor and adjacent nontumor tissues from 45 PC patients in GSE28735. (B) Kaplan–Meier survival curves of OS of the 182 PC patients in TCGA database according to *IDO1* and *SLC2A1* coexpression level. (C) Tumor volumes recorded every 2 days, *n* =  5/group. (D) Tumors weighed at the completion of treatments, *n* =  5/group. (E) Image of tumors. (F) Body weight was measured every 2 days, *n* =  5/group. (G) Spleen weighed at the completion of treatments, *n* =  5/group. (H) Trp and Kyn levels in serum analyzed by HPLC, *n* =  5/group. (I) Representative TUNEL stained images of the paraffin‐embedded tumor tissue sections from 50 sections from 5 mice/group and the scale bar is 50 µm. Data in panel (A) were analyzed by the Student's *t*‐test, data in panels (C) and (F) were analyzed by two‐way ANOVA with Bonferroni post hoc test, and data in panels (D) and (G)–(I) were analyzed by one‐way ANOVA followed by Dunnett's post hoc test and expressed as mean ± SD, *p < 0.05, **p < 0.01, ***p < 0.001, ****p < 0.0001.

As shown in the above‐mentioned data, we demonstrated that IDO1 overexpression promotes glycolysis and decreases the apoptosis of PC cells, and IDO1 inhibitor inhibits glycolysis in PC. Herein, we further investigated the association between glycolysis and IDO1 in PC with bioinformatic analysis and found the expression of *SLC2A1*, *SLC2A3*, *SLC2A5* (encoding GLUT1, GLUT3, GLUT5 protein), and *IDO1* in the tumor tissues of PC patients was increased compared to the adjacent normal tissues (Figures 6A and S8). Analyzing the OS data of PC patients in The Cancer Genome Atlas (TCGA) database revealed that the OS of the *SLC2A1*‐high and *SLC2A10*‐high group was shorter than that of the low expression group (Figure S9). Notably, patients with high expression of both *IDO1* and *SLC2A1* (*IDO1*/*SLC2A1*‐high) had the shortest OS, while those with low expression of both *IDO1* and *SLC2A1* (*IDO1*/*SLC2A1*‐low) had the most prolonged OS among the four groups (Figure 6B). These findings suggest that the expression of *IDO1*/*SLC2A1* may be an independent prognostic indicator for PC, and inhibiting of IDO1 and GLUT1 with inhibitors may offer potential benefits for PC patients.

We then explored the potential therapeutic efficacy of IDO1 inhibitors and/or GLUT1 inhibitors in PC mice. To this end, we used the canonical IDO1 inhibitor 1‐methyl‐l‐tryptophan (1‐MT) and the selective GLUT1 inhibitor BAY876. Using the Pan02 tumor‐bearing PC mice, we found that BAY876 exerted a modest inhibition on tumor growth, while 1‐MT resulted in a more effective suppression (Figures 6C–E) without apparent toxicity (Figures 6F and G). Notably, the combination of 1‐MT and BAY876 (combo) exhibited the most potent tumor growth inhibition among the four groups (Figures 6C–E). In addition, 1‐MT significantly suppressed IDO1 activity (reflected by the ratio of Kyn/Trp^44^) in serum (Figure 6H). Further, terminal deoxynucleotidyl transferase dUTP nick end labeling (TUNEL) staining of tumor tissue sections showed that 1‐MT had a proapoptotic effect while the combo led to a more robust proapoptotic effect than the monotherapy (Figure 6I). Some IDO1 inhibitors alone have shown only moderate therapeutic efficacies in animal models of PC,^45^, ^46^, ^47^ the exploration of new combination therapies is a promising strategy to promote IDO1‐based therapies from bench to bedside. Our data may provide a rationale for therapy with combinations of IDO1 inhibitors and GLUT1 inhibitors against PC.

## 3 DISCUSSION

Since IDO1's role in immune tolerance had been distinguished in 1998,^48^ the metabolic function of the enzyme was unappreciated. Recent research from Soraya Taleb's laboratory revealed IDO1's unexpected function in metabolic disorders, which brought our eyes back to the metabolic function of IDO1 in metabolic disease.^2^ Research on the effects of IDO1 on glycolysis in T and embryonic stem cells^7^, ^9^, ^10^, ^11^ focused our attention on the possible IDO1‐regulated glycolysis in PC, which was also considered a highly metabolic disease.^21^, ^22^ To this end, the positive correlation between the expression of *IDO1* and genes encoding rate‐limiting glycolytic enzymes in PC patients was revealed with bioinformatic tools, the regulatory effect of IDO1 on glycolysis was confirmed in mouse and human PC cells, and the inhibitory effect of IDO1 inhibitors on glycolysis was demonstrated in *vivo*.

To our knowledge, this study reveals for the first time the regulatory effect of cancer cell‐expressed IDO1 on glycolysis. Previous bioinformatic analyses have examined the IDO1–glycolysis link in tumor tissues,^49^, ^50^ yet they fell short of concretely defining the role of cancer cell‐expressed IDO1 in glycolysis. This gap in understanding is significant, considering that both immune and tumor cells can express IDO1. Furthermore, studies on mesenchymal stem cells cocultured macrophages noted simultaneous increases in IDO1 and GLUT1 expressions,^51^ without establishing a direct connection between the two. Contrastingly, research in the context of graft‐versus‐host disease identified a direct link between IDO1 and GLUT1,^52^ but it focused on how glycolysis influences IDO1, not vice versa. Moreover, although a limited body of studies have revealed the effects of T cell and embryonic stem cell‐expressed IDO1 on glucose metabolism,^7^, ^9^, ^10^, ^11^ these studies have not delved into the influence of IDO1 on glucose metabolism specifically within the tumor setting. Collectively, our study is the first to delineate how IDO1, when expressed by cancer cells, modulates glycolysis, filling a previously unexplored gap regarding IDO1's distinct impact on glucose metabolism across different cell types and pathological states, especially within the intricate tumor microenvironment.

Furthermore, the regulatory effect of IDO1 on glucose uptake, a rate‐limiting step of glycolysis, was found in PC cells. The bioinformatic analysis of the correlation between the expressions of *GLUT* family members and *IDO1* indicated the potential role of GLUT1 in IDO1‐modulated glucose uptake. And the effect of IDO1 on GLUT1 translocation to the PM was confirmed. We then determined experimentally that IDO1‐overexpression‐induced Trp deficiency enhanced glucose uptake by promoting GLUT1 translocation to the PM of PC cells. However, the underlying mechanism remains unknown. A recent study has shown that RhoA/ROCK plays a vital role in promoting the translocation of GLUT1 to the PM and then enhancing glucose uptake of cancer cells.^16^ Our future work will focus on whether RhoA/ROCK is key signaling regulating the IDO1‐involved translocation of GLUT1 in PC cells.

Aerobic glycolysis is a fundamental metabolic alteration that cancer cells undergo to impede apoptosis.^15^, ^32^, ^53^ We found IDO1‐overexpression‐induced Trp deficiency suppressed apoptosis of PC cells, which could be abolished by the block of glycolysis with GLUT1 inhibitor BAY876. IDO1 inhibitor RY103, Incyte, or 1‐MT promoted the apoptosis of PC cells in *vitro* and in *vivo*. Together, IDO1‐overexpression‐induced Trp deficiency suppressed the apoptosis of PC cells via promoting glycolysis. Our study revealed the presence of the IDO1–glycolysis–apoptosis axis in PC.

With the bioinformatics analysis in this study, we found the expression of *SLC2A1* (encoding GLUT1 protein) in PC tumors was significantly higher than adjacent normal tissues, and PC patients with high expression of *SLC2A1* had a worse prognosis, which is consistent with the previous studies.^54^, ^55^, ^56^ This suggests that GLUT1 is a promising drug target for PC. However, our data showed that GLUT1 inhibitor BAY876 only had moderate therapeutic efficacy in Pan02 tumor‐bearing PC mice. The bioinformatics analysis also showed that PC patients with high expression of *IDO1*/*SLC2A1* had a worse prognosis in comparison with those with low expression of these genes. We then proposed that IDO1 inhibitors may enhance the therapeutic efficacy of GLUT1 inhibitors in PC. Using the PC mice, robust therapeutic efficacy was found when IDO1 inhibitor 1‐MT was combined with GLUT1 inhibitor BAY876. Our current study provides a rationale for therapy with combinations of IDO1 inhibitors and GLUT1 inhibitors against PC and may hold potential implications for clinical applications.

**FIGURE 7:**
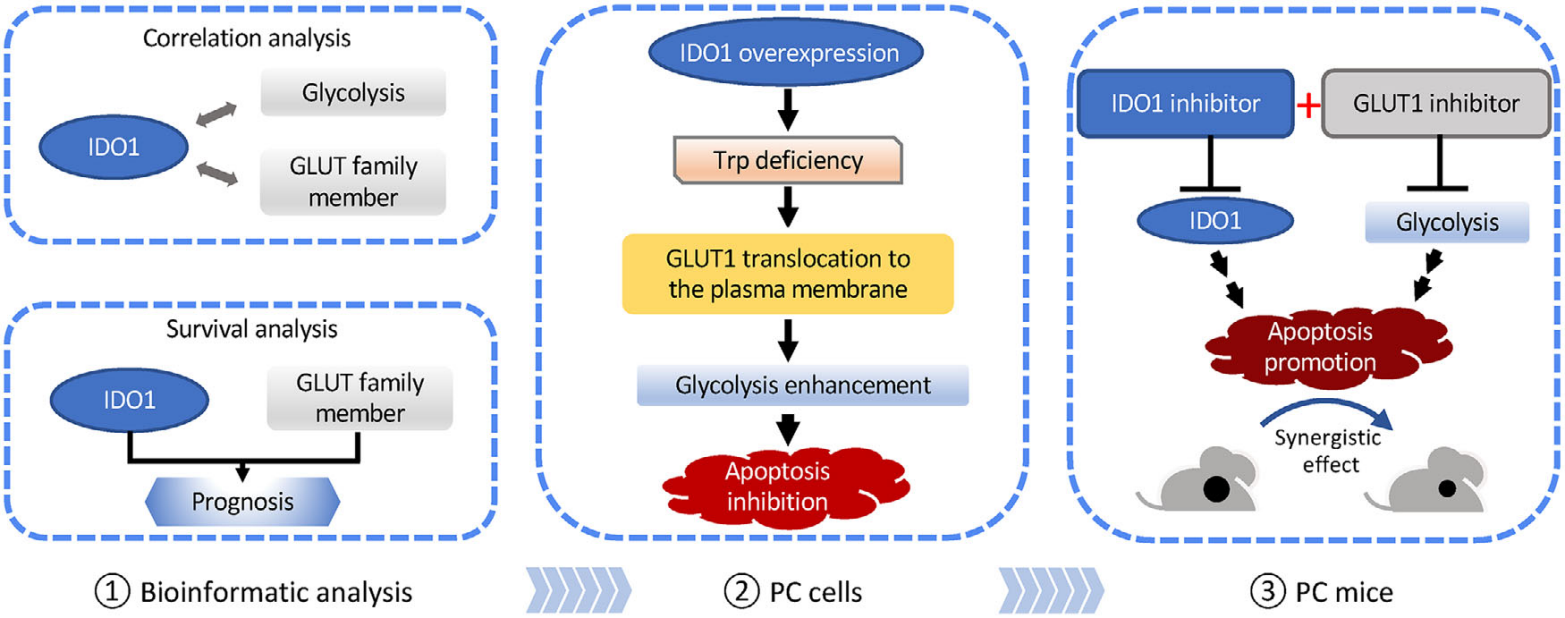
A schematic overview of IDO1‐induced tryptophan deficiency results in GLUT1‐dependent upregulation of glycolysis in pancreatic cancer.

Following a recent late‐stage clinical trial failure of Incyte (an IDO1 inhibitor) in combination with pembrolizumab (an anti‐PD1 antibody) in patients with advanced malignant melanoma,^57^ researchers are exploring the multiple and complex roles of IDO1 in disease. Our study reveals the function of IDO1 in the glucose metabolism of PC (for schematic overview see Figure 7) and may contribute to the growing knowledge base of IDO1 as well as the metabolic interactions of amino acids and glucose. Our discoveries may offer fresh perspectives on the pathogenesis and therapeutic approaches for PC.

## 4 MATERIALS AND METHODS

### 4.1 Patient clinical dataset download and analysis

The GSE28735 dataset was obtained from GEO. The data set comprises Pearson correlation analysis and *p* values for each gene comparison. It was employed to pinpoint genes correlated with *IDO1* expression in patients with PC. Genes with a Pearson correlation value of equal to or less than −0.25 (indicating a negative correlation) or equal to or greater than 0.25 (indicating a positive correlation), along with a *p* value less than 0.05, were singled out for further analysis.

The PC cases’ clinical information and mRNA sequencing data were downloaded through TCGA database. The Kaplan–Meier method was employed to calculate the OS rates over time, and the log‐rank test was employed to evaluate differences. The expression levels of *IDO1* and *SLC2A* family members were dichotomized into groups with high‐expression and low‐expression based on the mean value, respectively.

### 4.2 Plasmids

The mCherry–EGFP–GLUT1 plasmid was kindly provided by Dr. Huantian Zhang and Zhenyan Li (The First Affiliated Hospital, Jinan University). The human and mouse transient IDO1 expressing plasmids pcDNA3.1(+)–hIDO1 and pcDNA3.1(+)–mIDO1 were created by inserting full‐length human and mouse IDO1 cDNA between the *Kpn*I and *Not*I sites (for human) and the *Kpn*I and *Eco*RI sites (for mouse) of the pcDNA3.1(+) vector, respectively. The human and mouse stable IDO1 expressing plasmids pHBLV–CMV–MCS–3FLAG–EF1–ZsGreen–T2A–PURO–hIDO1 (pHBLV–hIDO1, constructed by Hanbio Biotechnology) and pBABE–mIDO1 were created by inserting full‐length human and mouse IDO1 cDNA between the *Bam*HI and *Eco*RI sites of the vector.

### 4.3 Cell culture

PANC1 and Pan02 cells were obtained from the Shanghai Cell Bank of the Chinese Academy of Sciences in China. The cells were cultured in DMEM (Gibco, USA) with 10% FBS (Gibco), 100 U/mL penicillin, and 100 µg/mL streptomycin (Gibco). Culturing was carried out in a humidified incubator set at 37°C with 5% CO_2_.

### 4.4 Transfection and retroviral infection

The pcDNA3.1(+)–hIDO1, pcDNA3.1(+)–mIDO1, and mCherry–EGFP–GLUT1 plasmids were transfected into PC cells using Lipofectamine 2000 Reagent (Invitrogen, USA) following the standard procedure.

Retroviruses carrying pHBLV–hIDO1 or pBABE–mIDO1 plasmid were generated in HEK293T cells with VSVG and GAG as packaging plasmids. The retroviral supernatant was collected 2 days after the initial plasmid transfection. Stable cell pools were established through a 2‐week selection with puromycin (Amresco, USA).

### 4.5 Reagents

RY103 is the IDO1 inhibitor designed and developed by our laboratory. 1‐MT (Cat number: 447439) was acquired from Sigma–Aldrich (Germany). INCB024360 (Incyte, Cat number: S7910) was purchased from Selleck (USA). Kyn (Cat number: K8625) was obtained from Sigma–Aldrich (Germany). BAY876 (Cat number: B422196‐1 mL and B287376‐10 mg) was purchased from Aladdin (China).

### 4.6 Cell treatment conditions

In transient overexpression experiments, PANC1 cells were transfected with pcDNA3.1(+)–hIDO1 plasmid for 48 or 72 h, Pan02 cells were transfected with pcDNA3.1(+)–mIDO1 plasmid for 48 h, cells transfected with empty plasmid pcDNA3.1(+) were used as control, and cells were incubated in a limited amount of cell culture medium.

In Trp deficiency experiments, PANC1 cells were incubated in customized Trp‐depleted DMEM (Cat number: CM0012X; Beijing Coolaber Technology, China) with 10% FBS for 72 or 6 h, Pan02 cells were incubated in customized Trp‐depleted DMEM with 10% FBS for 48 or 6 h, and cells cultured in DMEM supplemented with 10% FBS was used as control.

In Kyn treatment experiments, PANC1 and Pan02 cells were treated with 100 µM Kyn for 72 and 48 h, respectively. In IDO1 inhibitor treatment experiments, PANC1 and Pan02 cells were transfected with pcDNA3.1(+)–hIDO1 and pcDNA3.1(+)–mIDO1 plasmid and after transfection cells were immediately treated with 500 nM RY013 or 500 nM Incyte for 72 h or 48 h respectively, and IDO1 stable overexpressing Pan02 cells (pBABE–mIDO1) and PANC1 cells (pHBLV–hIDO1) were supplemented with 500 nM RY103 or 500 nM Incyte for 48 h and 72 h, respectively.

In GLUT1 inhibitor BAY876 treatment experiments, PANC1 and Pan02 cells were pretreated with 1 µM BAY876 for 12 h, and then incubated in Trp‐depleted DMEM with 10% FBS and 1 µM BAY876 for 6 h; PANC1 cells were transfected with pcDNA3.1(+)–hIDO1 for 72 h and Pan02 cells were transfected with pcDNA3.1(+)–mIDO1 plasmid for 48 h, both receiving 1 µM BAY876 treatment 24 h before the experiments ended.

### 4.7 Western blot assay

PC cells were lysed using the RIPA lysis buffer (Beyotime, China; Cat number: P0013B), and supernatants were collected as total protein. The PM fraction of cells was isolated following the standard procedure of membrane and cytosol protein extraction kit (Beyotime; Cat number: P0033). The protein concentration was assessed using the BCA Protein Assay Reagent (Beyotime; Cat number: P0012S). Lysates were subjected to 10% SDS‐PAGE and transferred onto PVDF membranes. Following blocking with PBS containing 5% nonfat milk, membranes were incubated with the respective primary antibodies: anti‐IDO1 (1:5000; Proteintech, USA; Cat number: 66528‐1‐Ig), anti‐HK2 (1:1000; Servicebio, China; Cat number: GB111063), anti‐LDHA (1:2000; Servicebio; Cat number: GB11342), anti‐GLUT1 (1:1000; Servicebio; Cat number: GB113495), anti‐Na+/K+ ATPase (1:1000; Beyotime; Cat number: AF1864), anti‐Bax (1:800; Servicebio; Cat number: GB114122), anti‐Bcl‐2 (1:1000; ABclonal, China; Cat number: A19693), or anti‐β‐actin (1:5000; Abways Technology, China; Cat number: AB2001) antibodies. After incubation with an HRP‐conjugated anti‐mouse secondary antibody (Beyotime) or anti‐rabbit secondary antibody (Beyotime), the proteins were detected using ECL reagents (Thermo Fisher Scientific, USA) and immunoreactive signals were quantitatively assessed using densitometry.

### 4.8 Metabolic studies

PK assay kit (Cat number: KTB1120), LDH assay kit (Cat number: KTB1110), and glucose assay kit (Cat number: KTB1300) were purchased from Abbkines (China). The glucose content detection kit (Cat number: BC2505) was procured from Beijing Solarbio Science & Technology Co., Ltd (China). All assays were performed according to the manufacturer's instructions.

### 4.9 Glucose uptake assay

Glucose uptake was performed by incubating the PC cells with a fluorescent probe 2‐NBDG (Cat number: 186689‐07‐6; Amgicam, China). Glucose uptake was measured by incubating the cells at 37°C for 20 min in glucose‐free DMEM (Gibco) containing 100 µM 2‐NBDG. Subsequently, the cells were harvested via trypsinization and then washed twice with ice‐cold PBS in the absence of light, and resuspended. The glucose uptake was quantified using Beckman Coulter's Gallios flow cytometer (Beckman, USA), and the data were analyzed using FlowJo software (Ashland, USA).

### 4.10 High‐performance liquid chromatography analysis of Trp and Kyn

The levels of Trp and Kyn in both serum and cell culture supernatant were determined using high‐performance liquid chromatography (HPLC), based on the retention time and the UV absorption (280 nm for Trp and 360 nm for Kyn). To eliminate proteins, serum or cell culture supernatant was treated with 5% perchloric acid and methanol. The supernatant was filtered through a 0.22 µm filter before undergoing HPLC analysis for Kyn and Trp. The analysis was carried out using the Agilent 1260 series HPLC system (Agilent Technologies, USA) equipped with a quaternary pump and a UV detector. The chromatographic conditions included a C18 column (250 mm × 4.6 mm, 5 µm; Agilent Technologies), a column temperature of 25°C, a mobile phase comprising 15 mM acetic acid–sodium acetate buffer (pH 3.6) with 6% acetonitrile by volume, a flow rate of 1 mL/min, and an injection volume of 20 µL.

### 4.11 Immunofluorescence staining

PC cells cultured on glass coverslips were fixed with 4% paraformaldehyde for 20 min. Subsequently, cells were permeabilized with 0.5% Triton X‐100 for 30 min. For GLUT1 staining, the fixed cells underwent incubation with GLUT1 antibody (1:100; Servicebio; Cat number: GB113495) at 4°C overnight following PBS washing. This succeeded by a 45‐min incubation with a goat anti‐rabbit secondary antibody (Alexa Fluor 488; 1:1000; Thermo Fisher Scientific; Cat number: A‐11034) after further PBS washing. DAPI staining (1:1000; Thermo Fisher Scientific) was then performed for 10 min. Slides were observed using a fluorescence microscope (FV3000; Olympus, Japan). Images were captured under consistent light conditions and exposure times.

### 4.12 Cell proliferation assay

PC cell proliferation was evaluated with the CCK‐8 Assay Kit (Cat number: FY600001‐5ML; Fuyuanbio, China) following the manufacturer's guidelines. The absorbance of each well was quantified using a Multiscan spectrum Mk3 (Thermo Fisher Scientific) at 450 nm.

### 4.13 Flow cytometry analysis of apoptosis

The AnnexinV‐FITC/PI double‐stained cell apoptosis detection kit (Cat number: FY600003‐100T; Fuyuanbio) was employed according to the manufacturer's instructions.

### 4.14 PC mice and treatment

The female C57BL/6 mice, aged 6–8 weeks, were procured from Shanghai Jiesijie Experimental Animal Co., Ltd (China), housed in a specific pathogen‐free vivarium, and fed standard chow.

*Pan02 orthotopic PC mice construction*: Pan02 cells were injected into the pancreas at 5×10^5^ per mouse. Three weeks later, tumor tissues were isolated and cut into little pieces that were intrapancreatic transplanted into mice. Fifteen days after tumor implantation, mice were randomly assigned, and treatments commenced on the same day. The treatment regimen is outlined as follows: Control, RY103, and Incyte group mice intraperitoneally (i.p.) received 10% 2‐hydroxypropyl‐β‐cyclodextrin (HPBCD) in normal saline every 36 h or every day, 6 or 36 mg/kg RY103 in 10% HPBCD every 36 h, and 50 mg/kg Incyte in 10% HPBCD every day, respectively. Mice were sacrificed after 2 weeks (36 mg/kg RY103 and 50 mg/kg Incyte) or 38 days (6 mg/kg RY103) of treatments. The spleen weight, tumor weight, and body weight were recorded.

*Pan02 tumor‐bearing mice construction*: Pan02 cells were subcutaneously injected into the right forelimb armpit at 2 × 10^6^ per mouse. Seven days postimplantation, the mice were randomly assigned, and treatments were initiated on the same day. The treatment regimen is outlined as follows: Control and 1‐MT group mice received 10% HPBCD and 100 mg/kg 1‐MT in 10% HPBCD, i.p., every 36 h, respectively. BAY876 group mice received 2.5 mg/kg BAY876 in 0.5% sodium carboxymethylcellulose (CMC‐Na), intragastrically (i.g.), every day. Combo group mice received 2.5 mg/kg BAY876 in 0.5% CMC‐Na, i.g., every day, and 100 mg/kg 1‐MT in 10% HPBCD, i.p., every 36 h. Body weight and subcutaneous tumor volumes were assessed every 2 days throughout the treatment period. Tumor dimensions were measured using vernier scale calipers, and the tumor volume was calculated using the formula: tumor size = long diameter × (short diameter)^2^/2. Mice were euthanized after 2 weeks of treatments, and the spleen weight, tumor weight, and body weight were documented.

### 4.15 Immunohistochemistry

Paraffin‐embedded tissue sections underwent dewaxing, rehydration, and antigen retrieval using citrate buffer. We then quenched endogenous peroxidase activity and blocked nonspecific binding with hydrogen peroxide and serum. The slides were incubated with anti‐LDHA (1:200; Servicebio; Cat number: GB11342) antibody overnight at 4°C. This was followed by the application of a secondary antibody from goat anti‐rabbit (Beyotime), visualization with DAB, and hematoxylin counterstaining. The H score was calculated as previously described.^58^

### 4.16 Analysis of biochemical parameters in serum

Glucose, cholesterol, HDL cholesterol, LDL cholesterol, triglycerides levels, and LDH activities were analyzed by ADVIA XPT (Siemens Healthcare Diagnostics, USA).

### 4.17 TUNEL assay

Sections of paraffin‐embedded tumor tissue were cut and affixed to slides. The TUNEL assay was conducted following the guidelines of the TUNEL kit (Cat number: G1502‐50T; Servicebio). DAPI (Cat number: C1002; Beyotime) was employed for nuclear staining. Subsequently, the slides were sealed with an antifluorescence quenching sealing solution and examined using a laser scanning confocal microscope (FV3000; Olympus). The percentage of apoptosis cells was quantified with ImageJ software.

### 4.18 Statistical analysis

Statistical analysis was conducted using GraphPad Prism 8. Student's *t*‐test, one‐way analysis of variance (ANOVA), and two‐way ANOVA analysis were utilized to assess statistical differences among groups. Comparisons between the two groups were performed using Student's *t*‐test, while comparisons across multiple groups were conducted through one‐way ANOVA. For comparisons across multiple groups and time points, a two‐way ANOVA test was employed. Results are presented as means ± standard deviation (SD) based on a minimum of three independent experiments. A significance level of *p* < 0.05 was considered statistically significant (*p < 0.05; **p < 0.01; ***p < 0.001; ****p < 0.0001). The “*n*” in the figure legends indicates the number of patients, mice, or independent cell culture preparations unless otherwise specified.

## AUTHOR CONTRIBUTIONS

*Investigation, data curation, writing—original draft*: Heng Liang. *Data curation and investigation*: Jiani Zhan, Yunqiu Chen, Zikang Xing, Zhen Ning Tony He, Yuying Liu, Xuewen Li, Yijia Chen, and Zhiyao Li. *Resources*: Chunxiang Kuang. *Data curation, writing—review and editing*: Dan Yang. *Funding acquisition, conceptualization, supervision, writing—review and editing*: Qing Yang. All authors have read and approved the final manuscript.

## CONFLICT OF INTEREST STATEMENT

The authors declare no conflict of interest.

## ETHICS STATEMENT

All animal procedures were approved by the Animal Ethics Committee of Fudan University, and experiments complied with ARRIVE guidelines (Ethics approval number: BE1803).

## Supporting information

Supporting Information

## Data Availability

The datasets utilized or examined in this study can be obtained from the corresponding author upon reasonable request.
