# Supplementary material for: Tryptophan deficiency induced by indoleamine 2,3‐dioxygenase 1 results in glucose transporter 1‐dependent promotion of aerobic glycolysis in pancreatic cancer
Source: MedComm (2020). 2024 May 3;5(5):e555. doi: 10.1002/mco2.555 (PMC11066657; doi:10.1002/mco2.555)

Additional File

Tryptophan Deficiency Induced by Indoleamine 2,3-Dioxygenase 1 Results in Glucose Transporter 1-Dependent Promotion of Aerobic Glycolysis in Pancreatic Cancer

Heng Liang[1], Jiani Zhan[1], Yunqiu Chen[1], Zikang Xing[1], Zhen Ning Tony He[1], Yuying Liu[1], Xuewen Li[1], Yijia Chen[1], Zhiyao Li[1], Chunxiang Kuang[2], Dan Yang[3]*, Qing Yang[1]*

[1] State Key Laboratory of Genetic Engineering, School of Life Sciences, MOE Engineering Research Center of Gene Technology, Shanghai Engineering Research Center of Industrial Microorganisms, Fudan University, Songhu Road 2005, Shanghai, 200438, China

[2] Shanghai Key Lab of Chemical Assessment and Sustainability, School of Chemical Science and Engineering, Tongji University, Siping Road 1239, Shanghai, 200092, China

[3] Department of Orthopedics, Shanghai Children's Hospital, School of Medicine, Shanghai Jiao Tong University, Luding Road 355, Shanghai, 200062, China

This file includes:

Supplementary figures

Figure S1. Overexpression of IDO1 in PC cells promotes glycolysis. ……………… 3

Figure S2. Trp deficiency induced by IDO1 overexpression enhances glucose uptake in PC cells. ……………………………………………………………………………… 5

Figure S3. Trp deficiency induced by IDO1 overexpression stimulates GLUT1 translocation to PM of PC cells. ……………………………………………………… 6

Figure S4. Trp deficiency induced by IDO1 overexpression inhibits apoptosis but does not affect the proliferation of PC cells. ········ 8

Figure S5. IDO1 inhibitor RY103 downregulates glycolytic enzyme expression in tumors and reduces LDH levels in the serum of orthotopic PC mice. ········ 9

Figure S6. IDO1 inhibitor Incyte downregulates glycolytic enzyme expression in tumors and reduces LDH levels in the serum of orthotopic PC mice. ········ 10

Figure S7. Long-term administration of RY103 suppresses tumor growth without apparent toxicity. ········ 11

Figure S8. Gene expression levels of SLC2A family in tumor and adjacent non-tumor tissues from 45 PC patients in GSE28735. ········ 12

Figure S9. Kaplan-Meier survival curves of OS of the 182 PC patients in TCGA database according to SLC2A family gene expression level. ········ 13

Uncropped scans of the Western blots ········ 14

Supplementary Figures

Figure S1

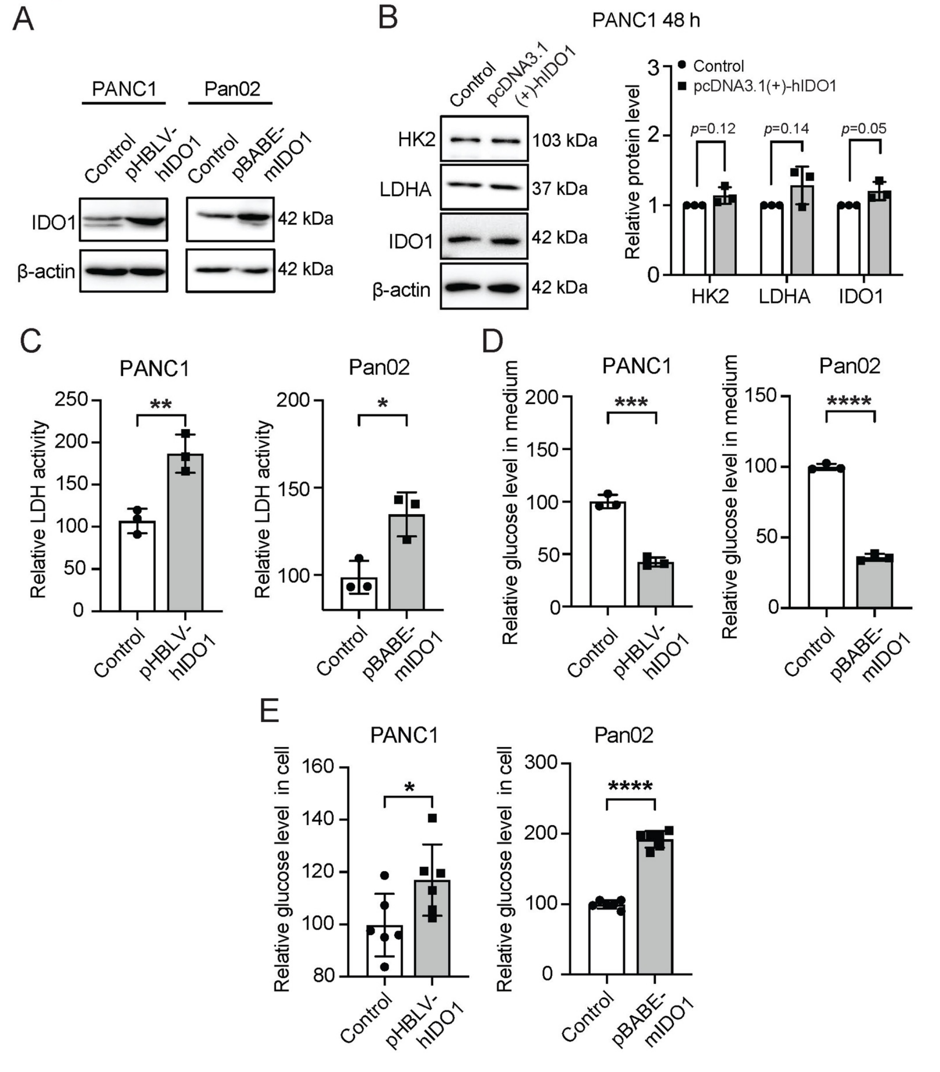

Figure S1. Overexpression of IDO1 in PC cells promotes glycolysis.

A IDO1 expression in IDO1 stable overexpressing PANC1 and Pan02 cells detected by Western blot. **B** Protein expression levels of HK2, LDHA, and IDO1 in PANC1 cells detected by Western blot. PANC1 cells were transfected with IDO1 expressing plasmids and then cultured for 48 h. **C-E** LDH activity and glucose level in IDO1 stable overexpressing Pan02 or PANC1 cells that grown for 48 or 72 h respectively. **C** LDH activity analyzed by colorimetric assay, n=3. **D** Glucose level in cell culture medium analyzed by colorimetric assay, n=3. **E** Glucose level in cells analyzed by

colorimetric assay, n=6. Data were analyzed by the Student's t-test and expressed as mean ± S.D., * $p < 0.05$, ** $p < 0.01$, *** $p < 0.001$, **** $p < 0.0001$.

Figure S2

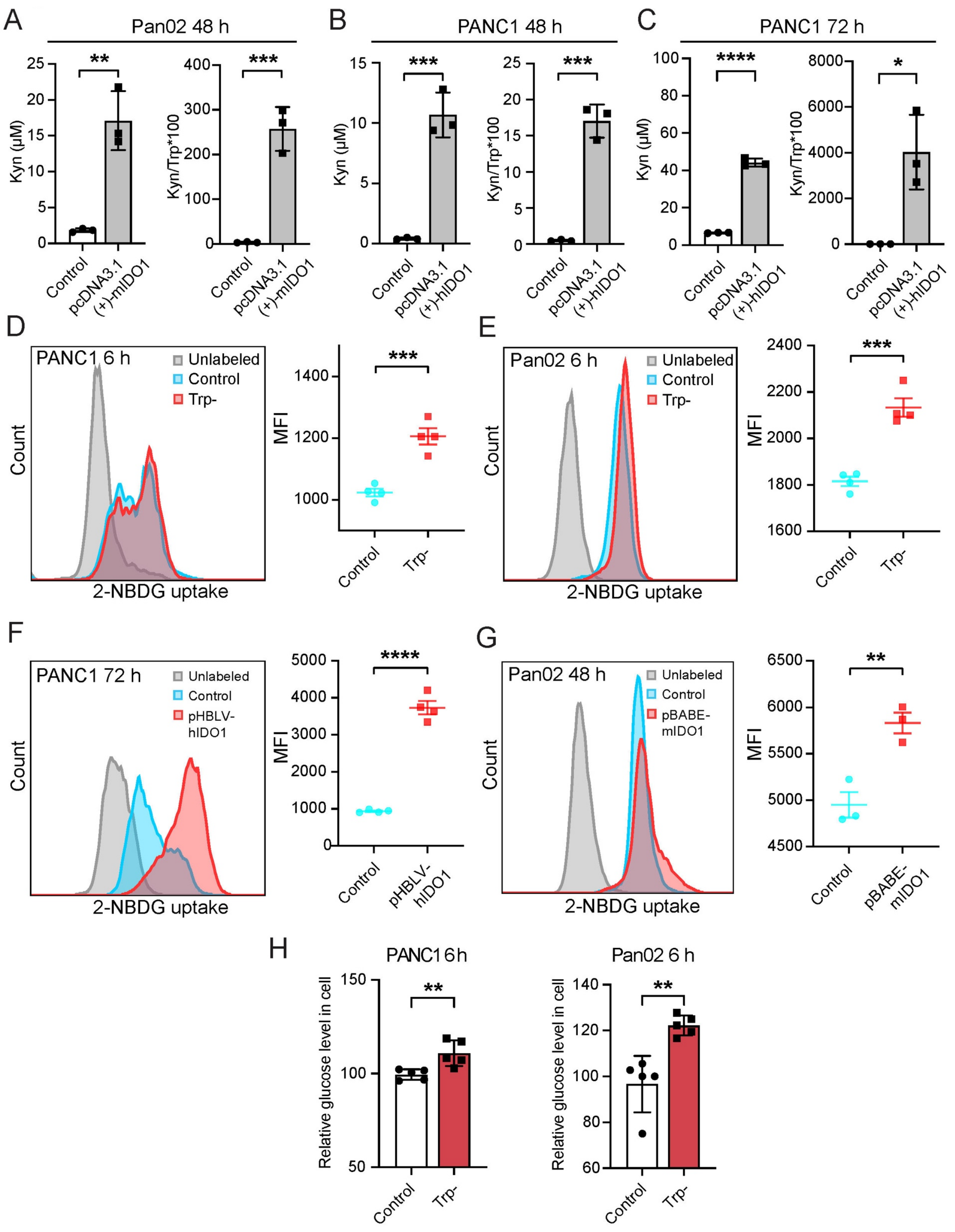

Figure S2. Trp deficiency induced by IDO1 overexpression enhances glucose uptake in PC cells.

A-C Effect of IDO1 overexpression on Kyn levels and IDO1 activity (Kyn/Trp) in PC cells. PC cells were transfected with IDO1 expressing plasmids and then cultured for 48 h (**A**, **B**) or 72 h (**C**).

Kyn levels in the supernatant of PC cells analyzed by HPLC, n=3. **D, E, H** Effects of Trp deficiency on glucose uptake (**D**, **E**) and glucose level (**H**) in PC cells. Trp-: PC cells cultured with Trp deficient medium for 6 h. Glucose uptake in PC cells determined using 2-NBDG by flow cytometry, n=4. Glucose level in PC cells analyzed by colorimetric assay, n=5. **F, G** Effects of IDO1 overexpression on glucose uptake in PC cells. IDO1 stable overexpressing PANC1 and Pan02 cells were cultured for 72 h and 48 h, respectively. Glucose uptake in PC cells determined using 2-NBDG by flow cytometry, n=3-4. One representative data from three independent assays was shown. Data were analyzed by the Student's t-test and expressed as mean ± S.D., * $p < 0.05$, ** $p < 0.01$, *** $p < 0.001$, **** $p < 0.0001$.

Figure S3

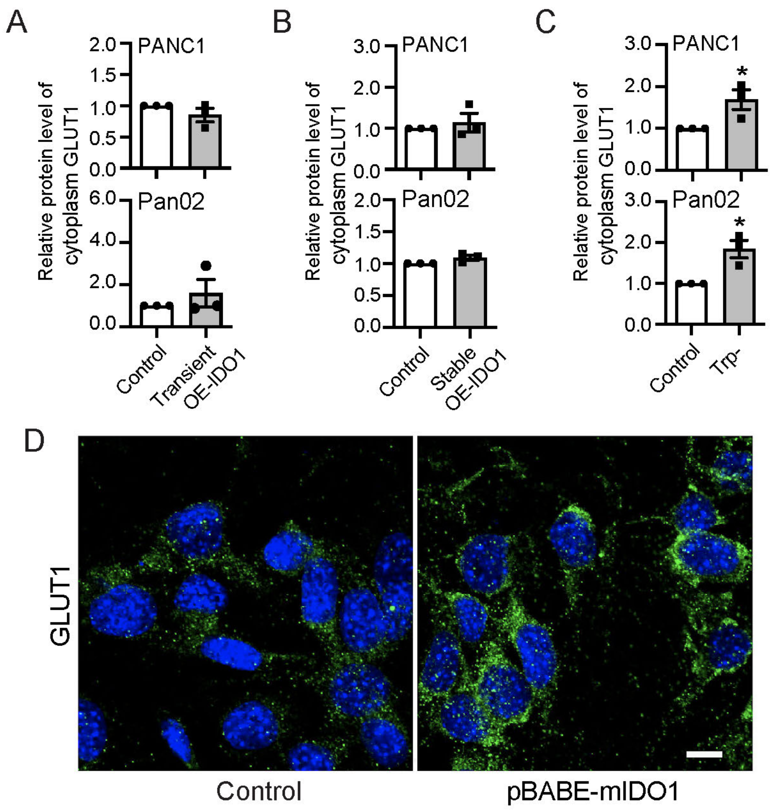

Figure S3. Trp deficiency induced by IDO1 overexpression stimulates GLUT1 translocation

to PM of PC cells.

A-C Quantification of the Western blot analysis of protein expression of cytoplasm GLUT1 in PC cells, β-actin was set as a loading control for cytoplasm protein. **A** PC cells were transfected with IDO1 expressing plasmids and then cultured for 48 h (Pan02) or 72 h (PANC1), n=3. **B** IDO1 stable overexpressing PC cells were cultured for 48 h (Pan02) or 72 h (PANC1), n=3. **C** Trp-: PC cells were cultured with Trp deficient medium for 6 h, n=3. **D** Effect of IDO1 overexpression on GLUT1 translocation to PM analyzed by immunofluorescence staining. IDO1 stable overexpressing Pan02 cells were cultured for 48 h on coverslips. GLUT1 (Green), nucleus (DAPI, Blue), scale bar: 10 μm. Data were analyzed by the Student's t-test and expressed as mean ± S.D., * $p < 0.05$.

Figure S4

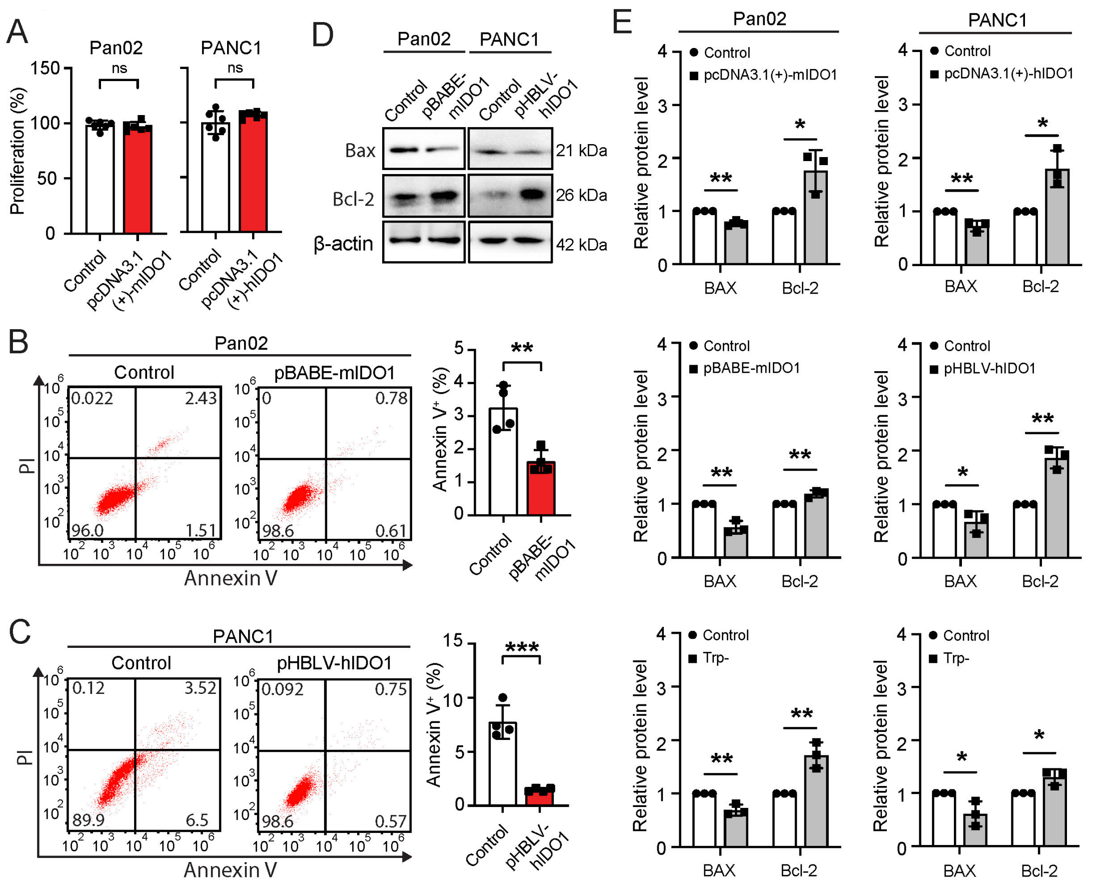

Figure S4. Trp deficiency induced by IDO1 overexpression inhibits apoptosis but does not affect the proliferation of PC cells.

A Effect of IDO1 overexpression on PC cell proliferation. PC cells were transfected with IDO1 expressing plasmids then cultured for 48 h (Pan02) or 72 h (PANC1). The proliferation of cells was assessed by CCK-8 Assay, n=6. **B-D** Effect of IDO1 overexpression on PC cell apoptosis. IDO1 stable overexpressing PC cells were cultured for 48 h (Pan02) or 72 h (PANC1). **B**, **C** Apoptotic cells were determined by flow cytometry with staining of AnnexinV/PI, n=4. **D** Protein expression of Bax and Bcl-2 in PC cells were detected by Western blot. **E** Effect of IDO1 expression and Trp deficiency on PC cell apoptosis. Protein expression levels of Bax and Bcl-2 in PC cells were detected by Western blot. Data were analyzed by the Student's t-test and expressed as mean ± S.D., ns: not

significant, * $p < 0.05$, ** $p < 0.01$, *** $p < 0.001$.

Figure S5

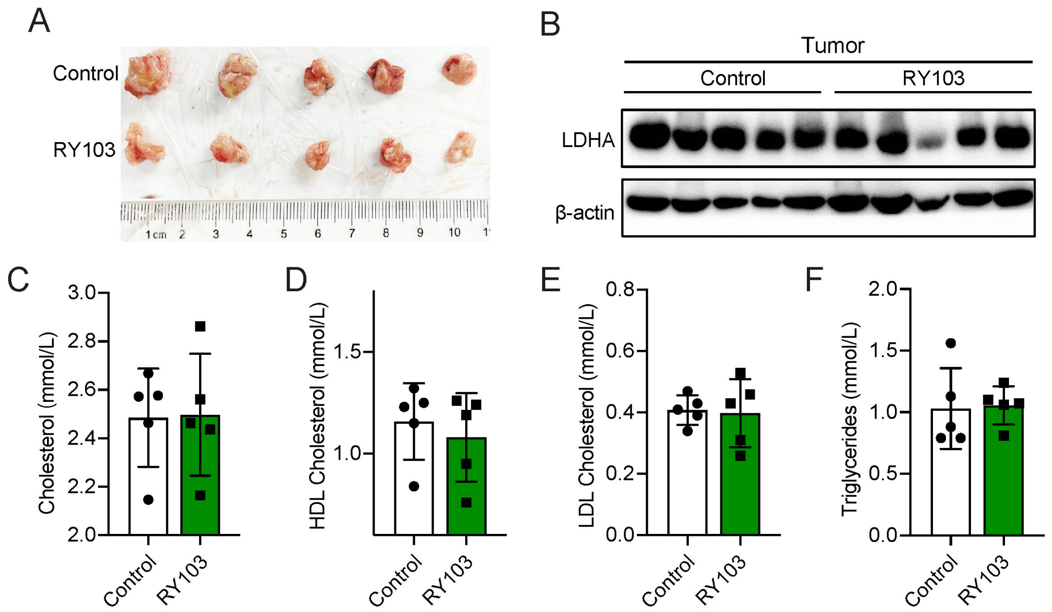

Figure S5. IDO1 inhibitor RY103 downregulates glycolytic enzyme expression in tumors and reduces LDH levels in the serum of orthotopic PC mice.

Pan02 orthotopic PC mice were administered with 36 mg/kg RY103 (i.p., every 36 h) for two weeks and sacrificed 24 h post the last administration. **A** Images of tumors derived from mice in each group. **B** Expression of LDHA in tumor tissues detected by Western blot, n=5/group. **C-F** Cholesterol, HDL cholesterol, LDL cholesterol, and triglycerides levels in serum analyzed by ADVIA XPT, n=5/group. Data were analyzed by the Student's t-test and expressed as mean ± S.D..

Figure S6

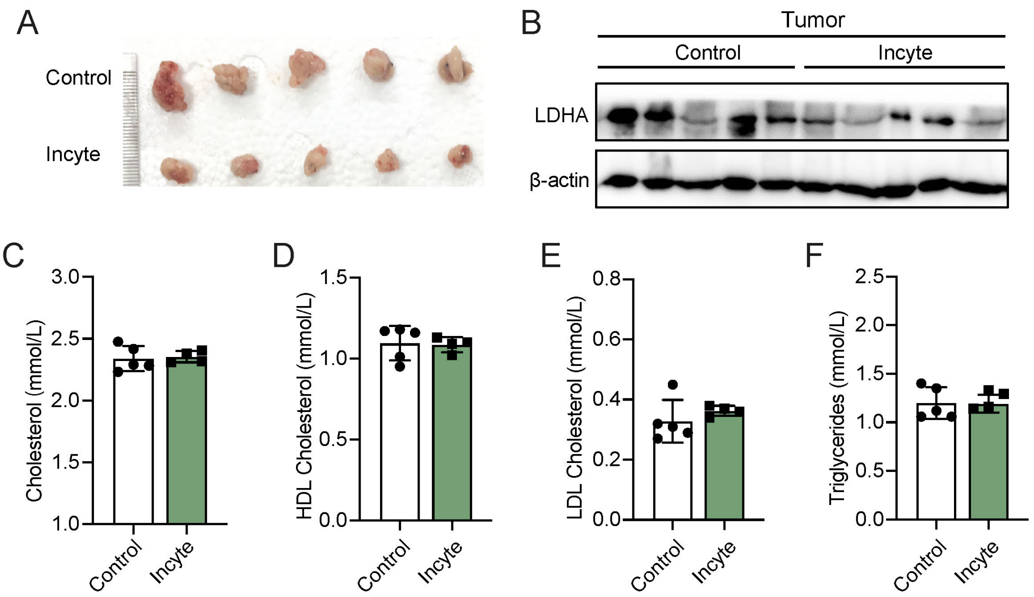

Figure S6. IDO1 inhibitor Incyte downregulates glycolytic enzyme expression in tumors and reduces LDH levels in the serum of orthotopic PC mice.

Pan02 orthotopic PC mice were administered with 50 mg/kg Incyte (i.p., every 24 h) for two weeks and sacrificed 24 h post the last administration. **A** Images of tumors derived from mice in each group. **B** Expression of LDHA in tumor tissues detected by Western blot, n=5/group. **C-F** Cholesterol, HDL cholesterol, LDL cholesterol, and triglycerides levels in serum analyzed by ADVIA XPT, Control: n=5/group, Incyte: n=4/group. Data were analyzed by the Student's t-test and expressed as mean ± S.D..

Figure S7

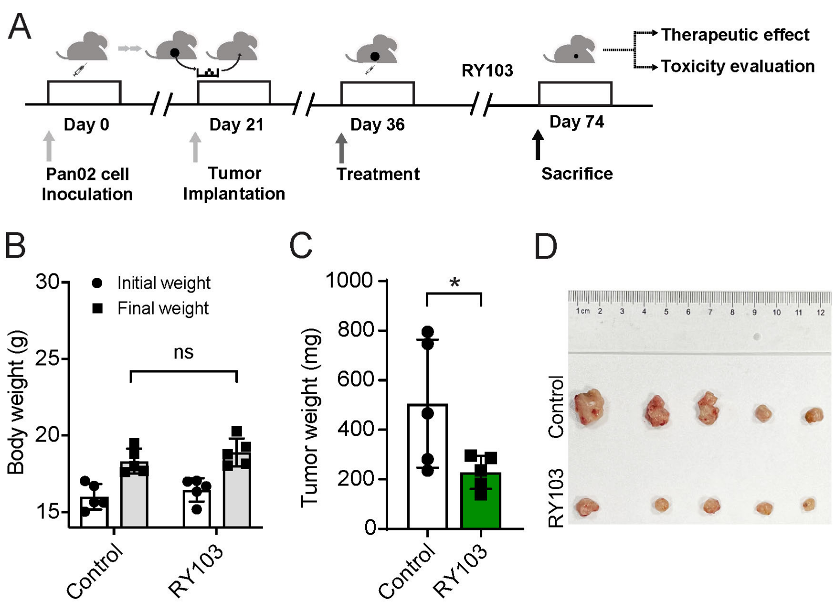

Figure S7. Long-term administration of RY103 suppresses tumor growth without apparent toxicity.

Pan02 orthotopic PC mice were administered with RY103 (6 mg/kg, i.p., every 36 h) for 38 days and sacrificed 24 h post the last administration. **A** Graphical representation outlining the treatment schedule. **B** Body weight measured at the initiation and termination of treatments. **C, D** Tumors isolated from each group of mice and weighed. n=5/group. Data were analyzed by the Student's t-test and expressed as mean ± S.D., ns: not significant, * $p < 0.05$.

Figure S8

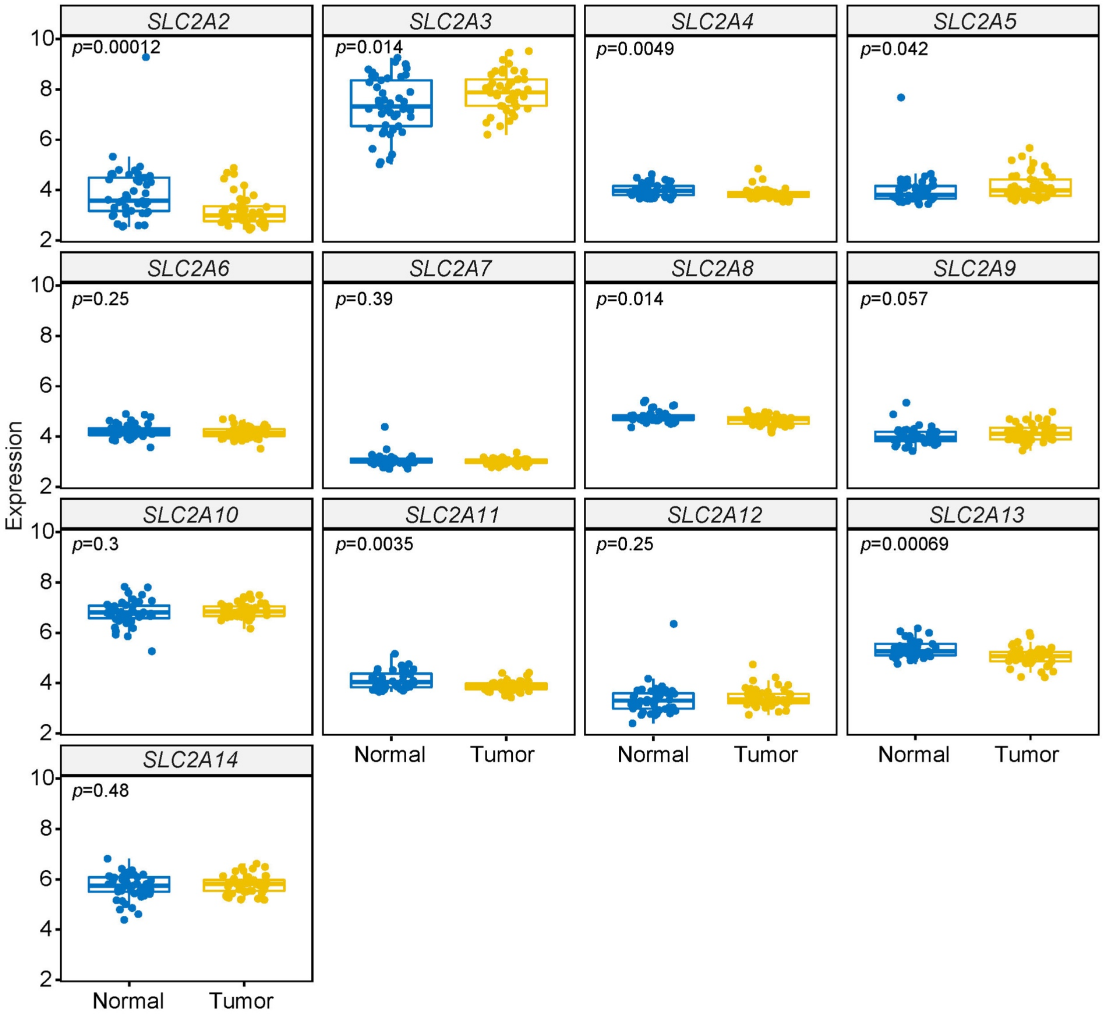

Figure S8. Gene expression levels of *SLC2A* family in tumor and adjacent non-tumor tissues from 45 PC patients in GSE28735.

Figure S9

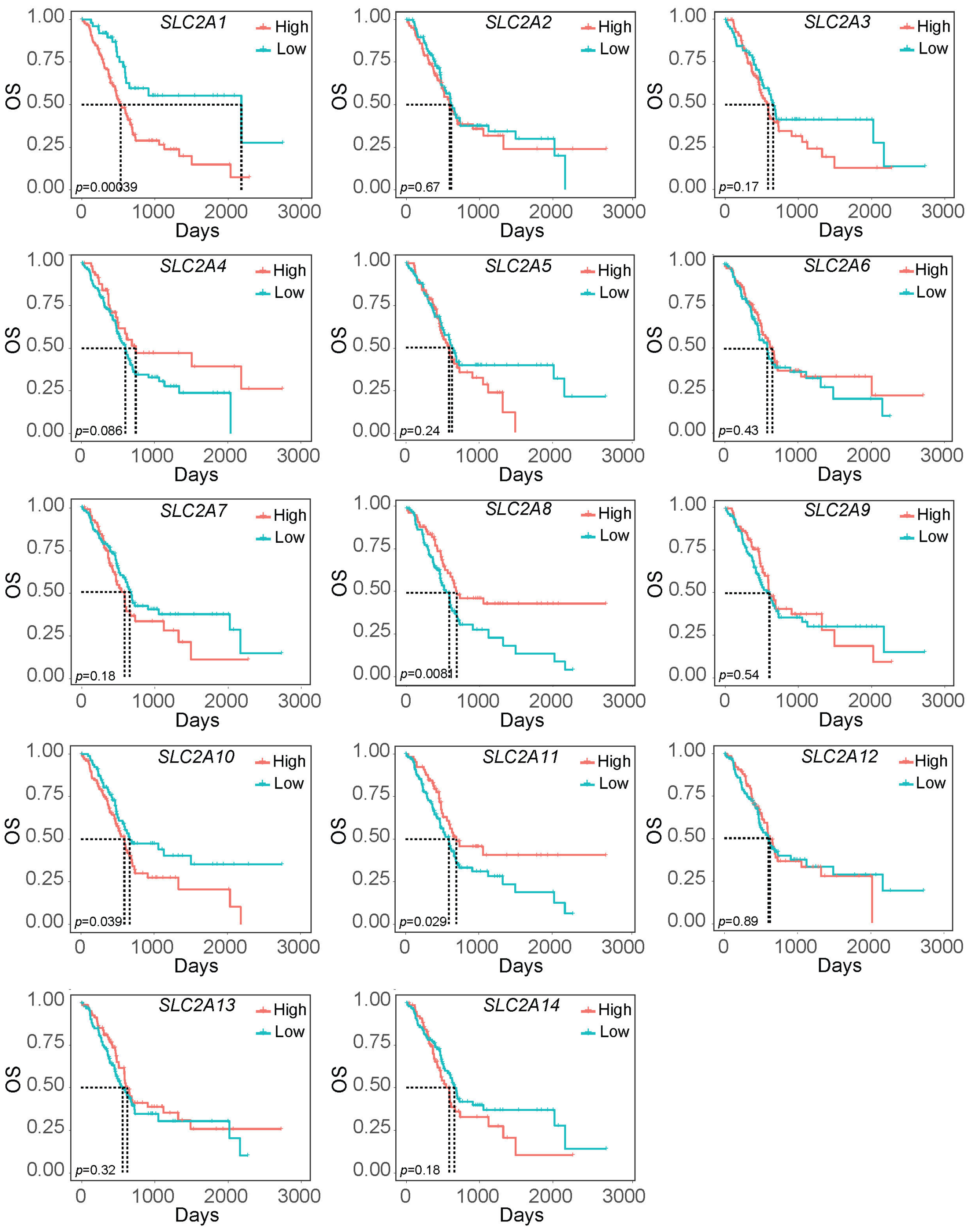

Figure S9. Kaplan-Meier survival curves of OS of the 182 PC patients in TCGA database according to *SLC2A* family gene expression level.

Uncropped scans of the Western blots

Uncropped scans of the Western blots used in Figure 1B:

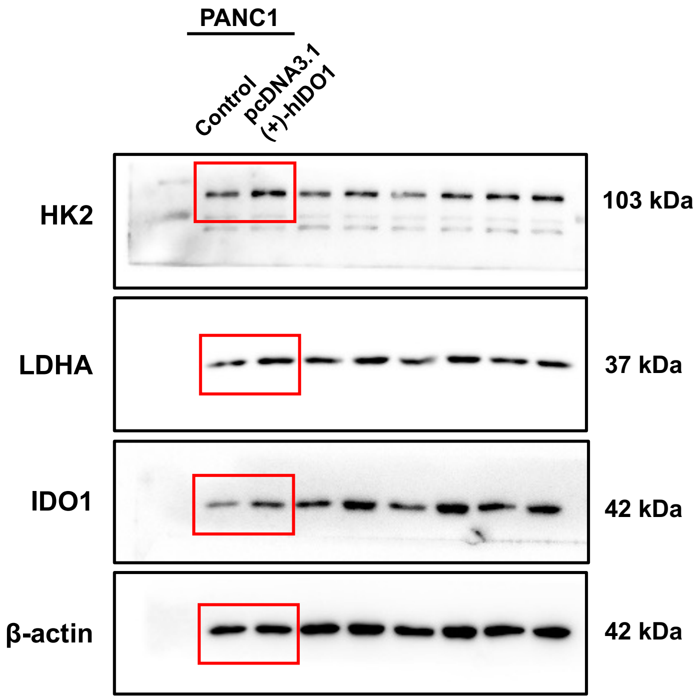

Uncropped scans of the Western blots used in Figure 1C:

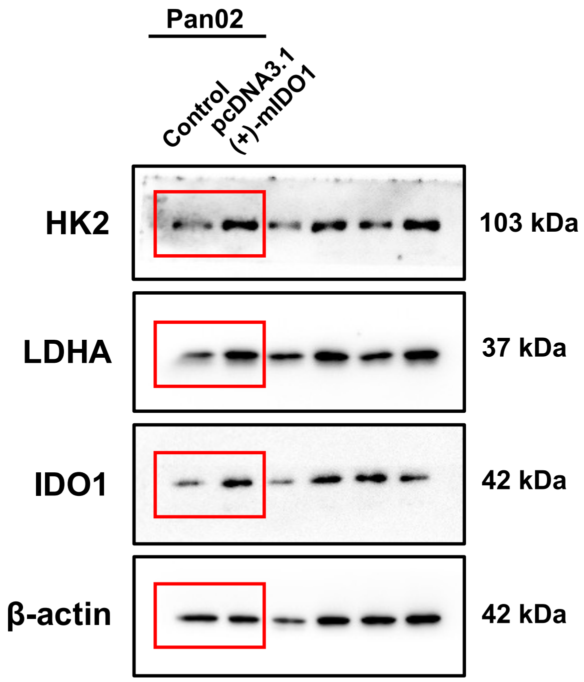

Uncropped scans of the Western blots used in Figure 3B:

Left:

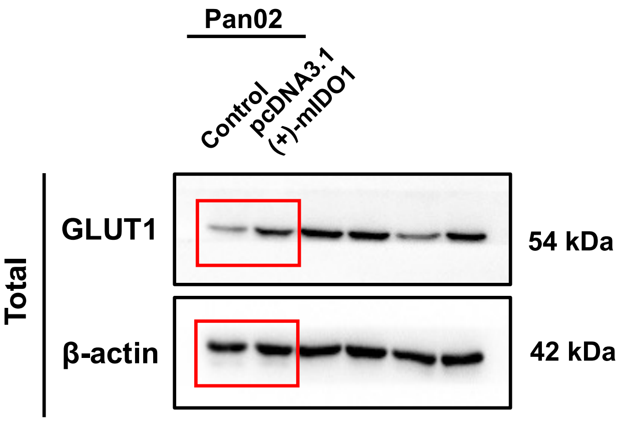

Right:

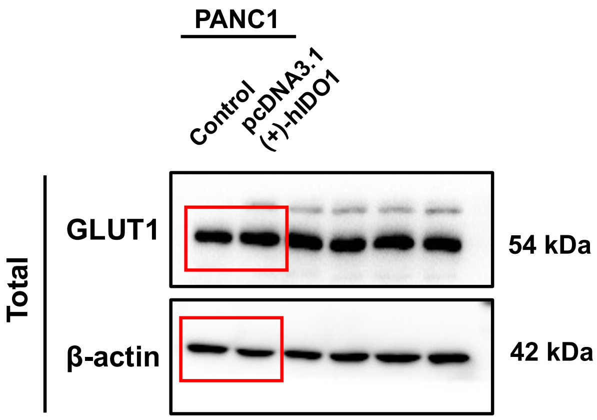

Uncropped scans of the Western blots used in Figure 3C:

Above:

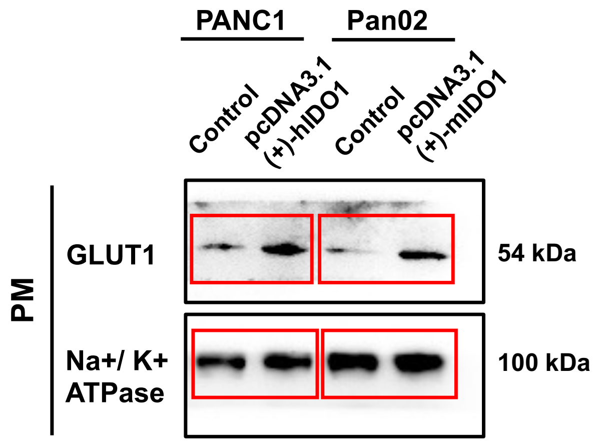

Down:

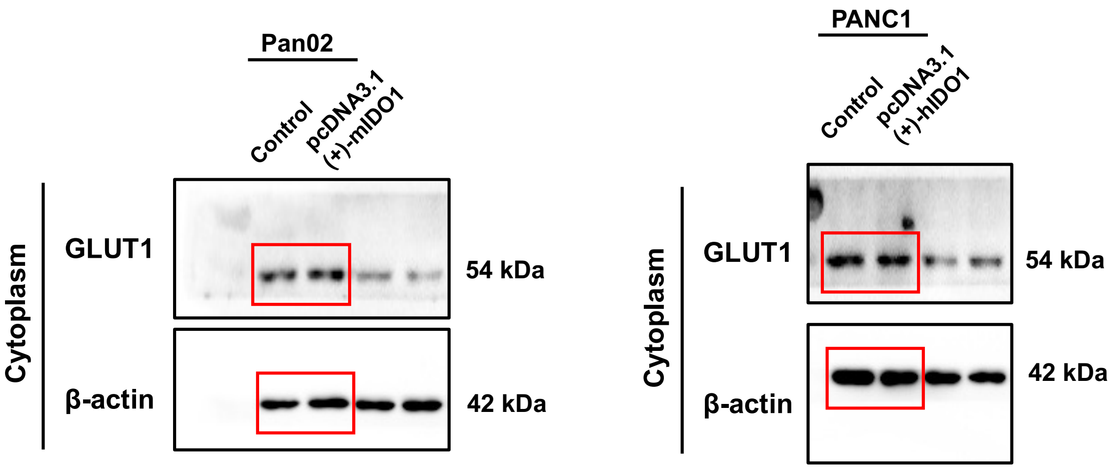

Uncropped scans of the Western blots used in Figure 3D:

Above:

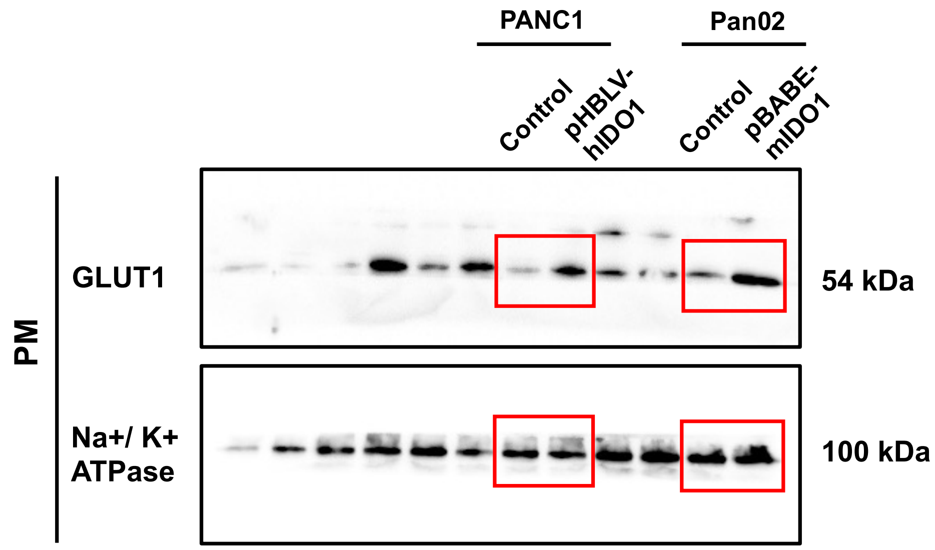

Down:

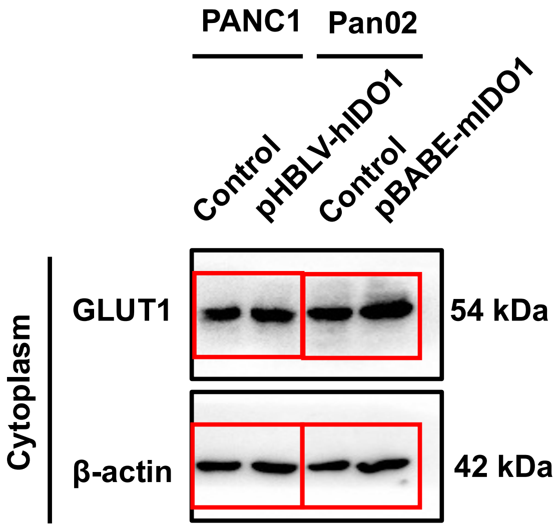

Uncropped scans of the Western blots used in Figure 3E:

Above:

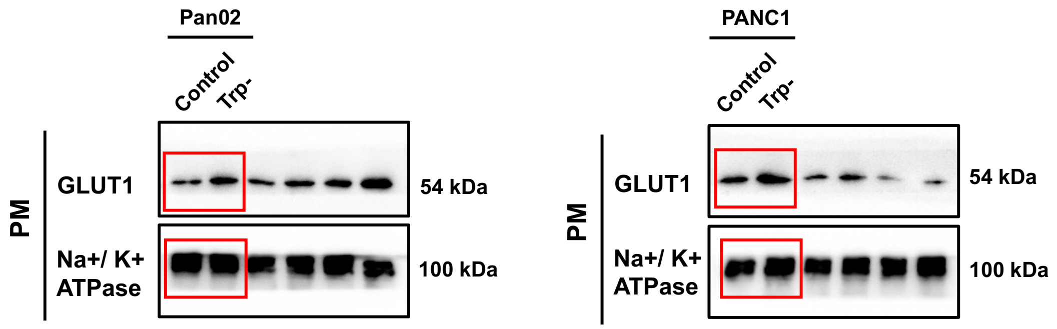

Down:

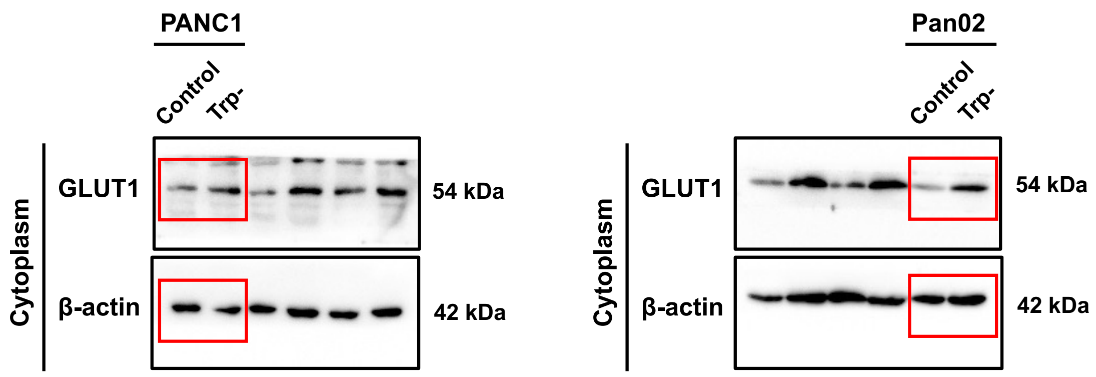

Uncropped scans of the Western blots used in Figure 3H:

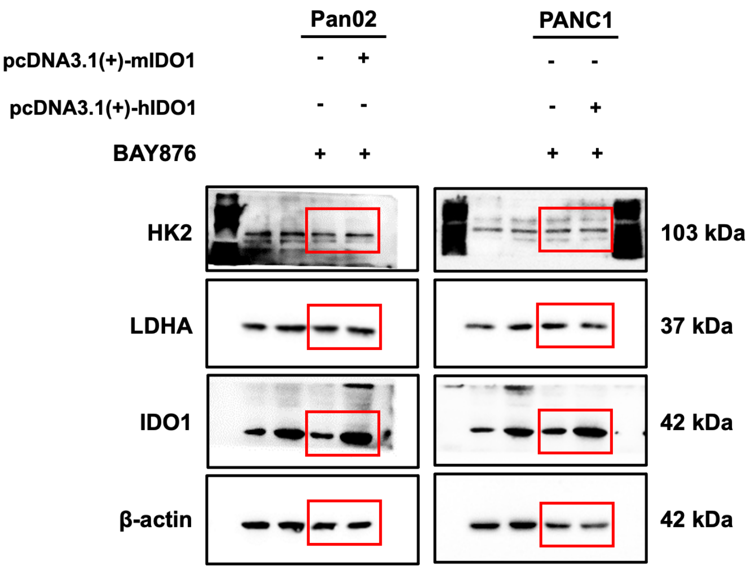

Uncropped scans of the Western blots used in Figure 4E:

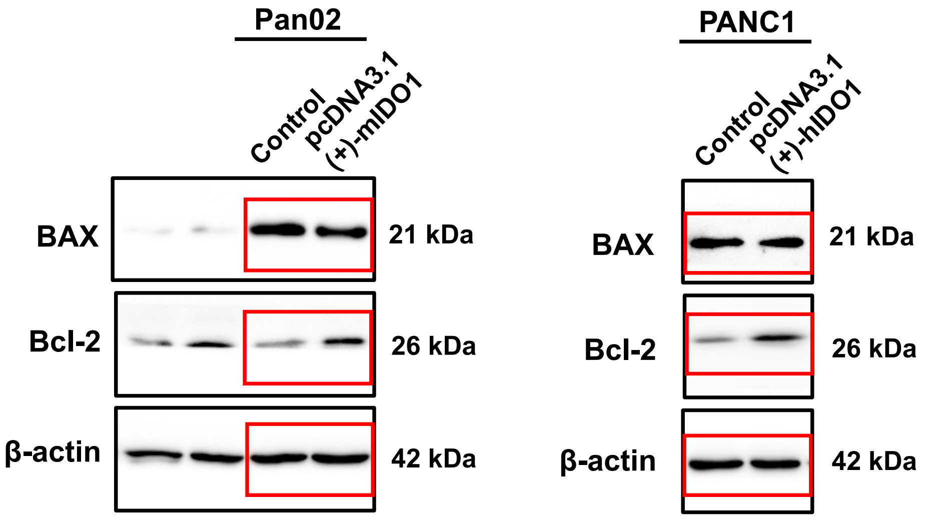

Uncropped scans of the Western blots used in Figure 4F:

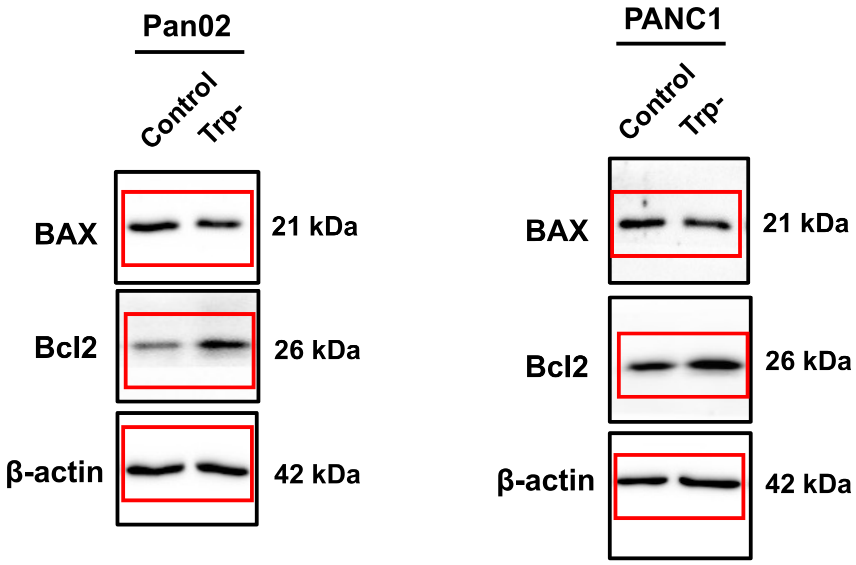

Uncropped scans of the Western blots used in Figure S1A:

Left:

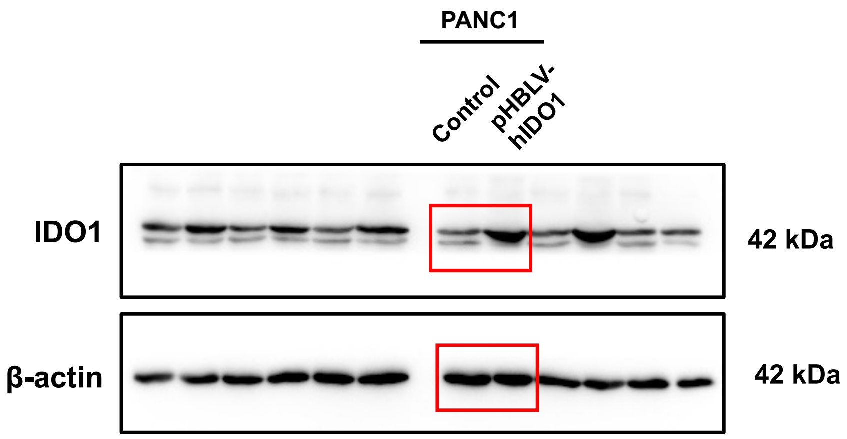

Right:

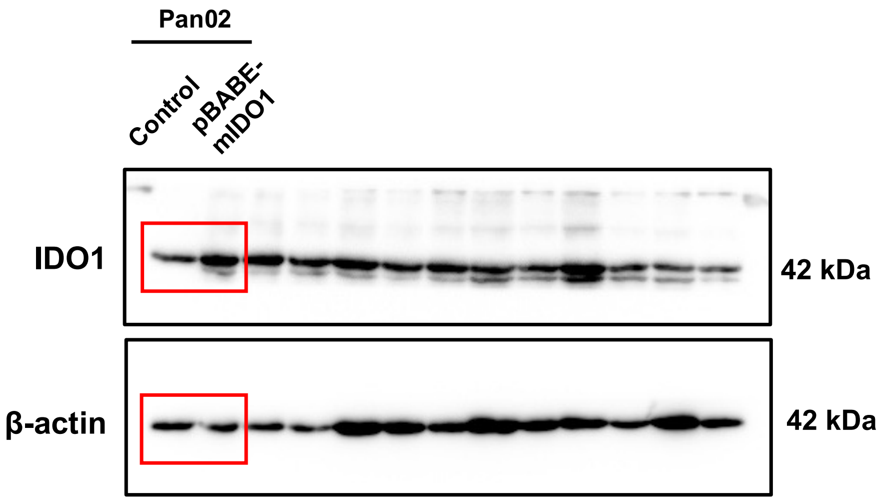

Uncropped scans of the Western blots used in Figure S1B:

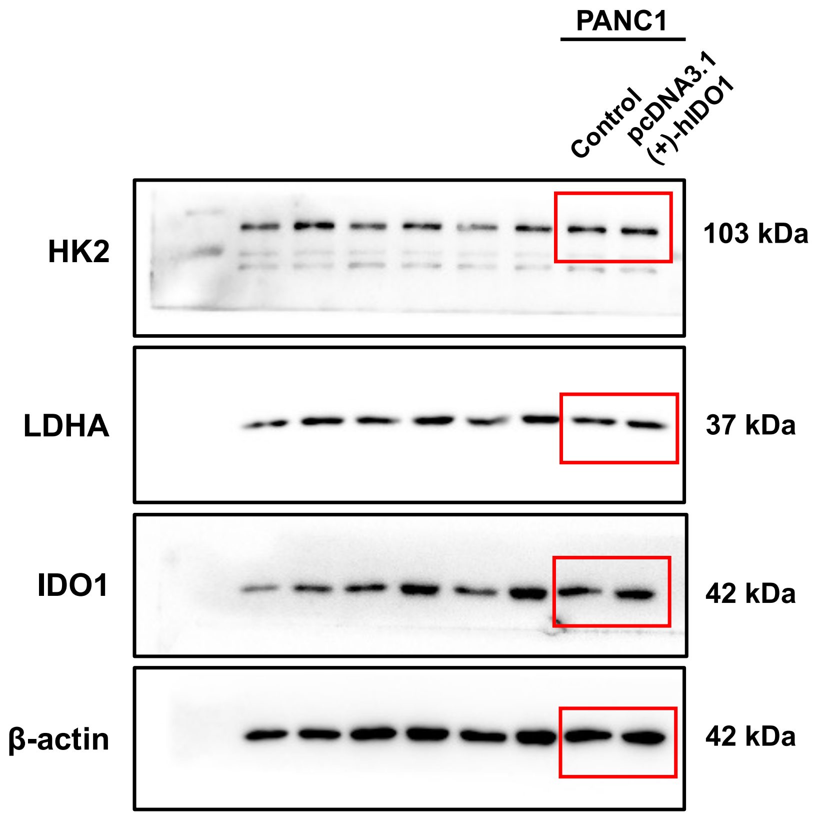

Uncropped scans of the Western blots used in Figure S4D:

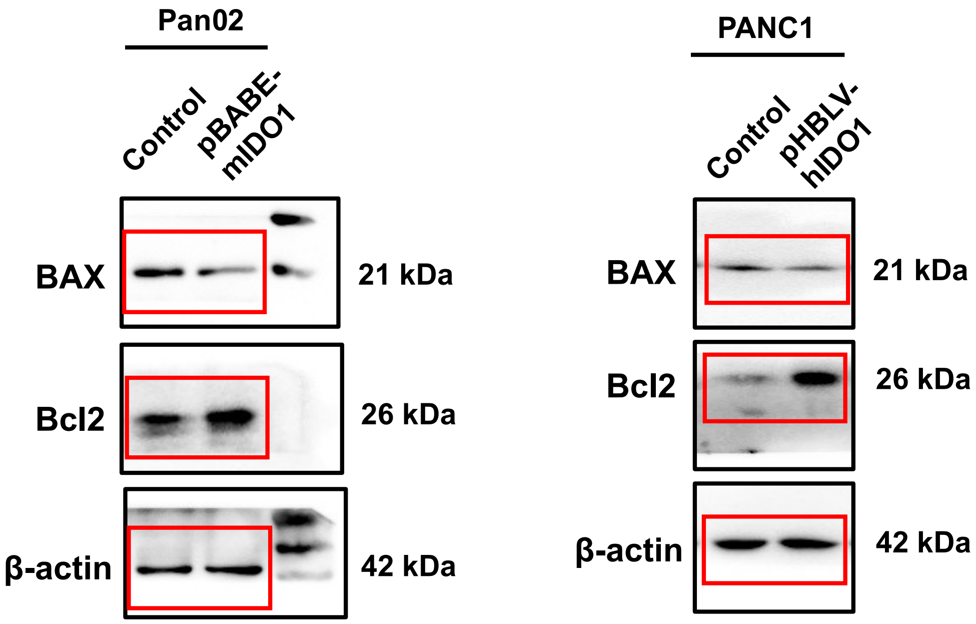

Uncropped scans of the Western blots used in Figure S5B:

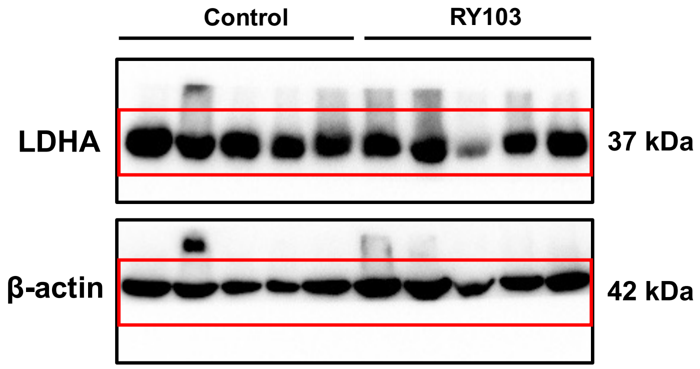

Uncropped scans of the Western blots used in Figure S6B:

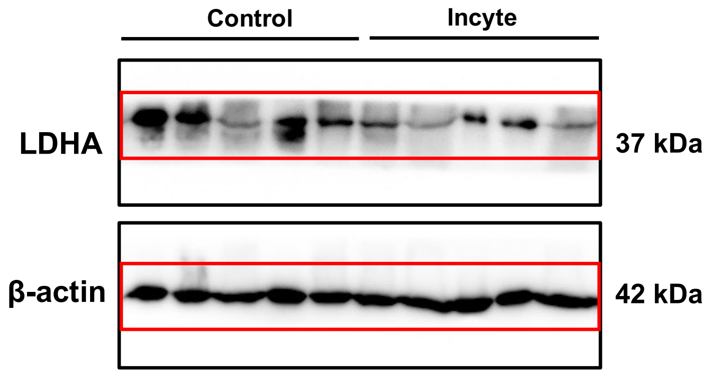

Supplement: Supplementary file 1 — Supporting Information [file MCO2-5-e555-s001.pdf]
